# G Protein‐Coupled Receptor in High‐Altitude Diseases: Mechanistic Insights and Therapeutic Opportunities

**DOI:** 10.1002/mco2.71033

**Published:** 2026-10-04

**Authors:** Qing Zhao, Changliang Liu, Lu Dai, Kun Zheng, Kun Liang, Chuanling Pan, Lianhua Liu, Ling Chen, Xuefei He, Hui Yan, Lei Chen, Huaping Zheng, Cheng Deng, Fengming Luo

**Affiliations:** ^1^ Department of High Altitude Medicine, Center for High Altitude Medicine, College of Life Sciences, National Clinical Research Center for Geriatrics, West China Hospital Sichuan University Chengdu Sichuan China; ^2^ Xi Zang Hospital of West China Hospital Sichuan University Lhasa Xizang China; ^3^ Harmful Components and Tar Reduction in Tobacco Key Laboratory of Sichuan Province Chengdu China; ^4^ Department of Intensive Care Unit, The Third People's Hospital of Chengdu Affiliated Hospital of Southwest Jiaotong University Chengdu China; ^5^ Center for High Altitude Medicine Xining China

**Keywords:** adaptation, GPCRs, high‐altitude disease, hypoxia, maladaptation

## Abstract

High‐altitude diseases (HADs) encompass a spectrum of acute and chronic disorders—including acute mountain sickness, high‐altitude cerebral edema, high‐altitude pulmonary edema, high‐altitude polycythemia, high‐altitude pulmonary hypertension, and high‐altitude heart disease—that arise from insufficient adaptation to hypobaric hypoxia. Despite their heterogeneous clinical manifestations, these conditions represent a continuum from acute adaptive stress to chronic maladaptive compensation, unified by persistent oxygen deprivation and dysregulated systemic homeostasis. G protein‐coupled receptors (GPCRs), the largest superfamily of membrane receptors, have emerged as central regulators of hypoxia sensing, ventilatory control, vascular tone, barrier integrity, erythropoiesis, immune activation, and metabolic reprogramming. However, how specific GPCR subtypes and their downstream signaling networks coordinate adaptation versus maladaptation across distinct organ systems and disease stages remains incompletely defined. This review synthesizes recent mechanistic insights into GPCR‐mediated pathways that govern neural, pulmonary, vascular, and hematologic responses to hypoxia. We examine the transition from protective to pathological signaling, highlight receptor‐level divergence that enables therapeutic selectivity, and evaluate translational prospects for GPCR‐targeted strategies, including biased agonism, receptor‐specific modulation, and sex‐based interventions. These advances establish a framework for precision therapies tailored to high‐altitude populations.

## Graphical abstract

High‐altitude hypobaric hypoxia activates multiorgan GPCR signaling networks that regulate ventilatory responses, cerebral endothelial barrier integrity, erythropoiesis and red blood cell metabolism, pulmonary vascular remodeling, and cardiac adaptation. Dysregulated GPCR pathways contribute to acute and chronic high‐altitude diseases, while clinically available agents, preclinical candidates, and genetic susceptibility factors provide opportunities for precision GPCR‐targeted prevention and therapy.

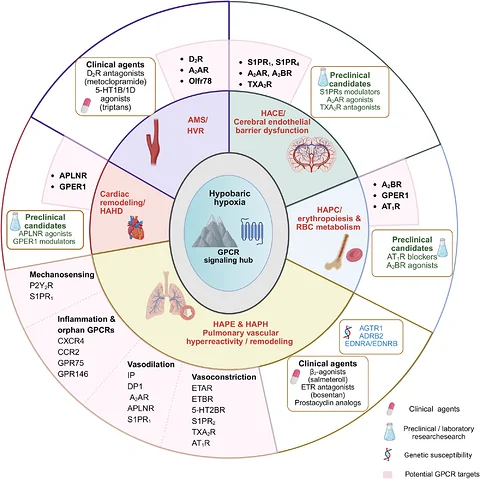

## 1 Introduction

High‐altitude diseases (HADs) are a spectrum of acute and chronic disorders triggered by exposure to hypobaric hypoxia [1]. HADs can be broadly classified into acute and chronic forms according to the timing of onset and clinical manifestations. Acute high‐altitude diseases (AHADs), including acute mountain sickness (AMS), high‐altitude cerebral edema (HACE), and high‐altitude pulmonary edema (HAPE), typically occur within a short period following ascent to high‐altitude environments [1]. AMS is the most common form of AHADs and usually develops shortly after ascent to high altitude [1]. Its incidence demonstrates a strong positive correlation with altitude. At 2850 m, the incidence rate is approximately 5.8%, while at higher elevations of 4559 m, this rate increases dramatically to 21.9% [2]. AMS typically resolves spontaneously, however, approximately 0.1%–2% of patients may progress to develop life‐threatening complications, including HAPE and HACE [3, 4, 5]. Chronic high‐altitude diseases (CHADs) encompass a spectrum of chronic maladaptive disorders caused by prolonged exposure to hypobaric hypoxia, including high‐altitude polycythemia (HAPC), high‐altitude pulmonary hypertension (HAPH), and high‐altitude heart disease (HAHD) [6, 7, 8]. These conditions constitute a major risk factor for myocardial infarction and stroke among high‐altitude populations [9]. The prevalence of CHADs exhibits considerable variation across different high‐altitude regions and ethnic groups. While Tibetan populations show a relatively low prevalence rate of 1.2%, Indigenous Andean communities experience significantly higher rates reaching up to 15% [10]. Current demographics indicate that China's high‐altitude regions house a permanent population exceeding 40 million residents. Additionally, over 100 million individuals annually travel to high‐altitude areas for tourism, occupational purposes, or athletic competitions. The manifestations of both AHADs and CHADs impose substantial economic and public health burdens on these regions, establishing this as a pressing public health challenge requiring immediate attention and comprehensive intervention strategies.

Despite their heterogeneous clinical manifestations, HADs share a common pathogenic origin in persistent hypobaric hypoxia [11, 12]. This oxygen deprivation disrupts vascular, inflammatory, and metabolic homeostasis across multiple organ systems, driving a spectrum of disease that ranges from acute adaptive stress upon rapid ascent to maladaptive compensations during sustained exposure [13, 14]. This review provides an integrated overview of the clinical features and disease progression across the spectrum of HADs, highlighting their shared hypoxic etiology and divergent clinical trajectories (Figure 1). In the following sections, we summarize these categories in detail, beginning with AHADs.

**FIGURE 1:**
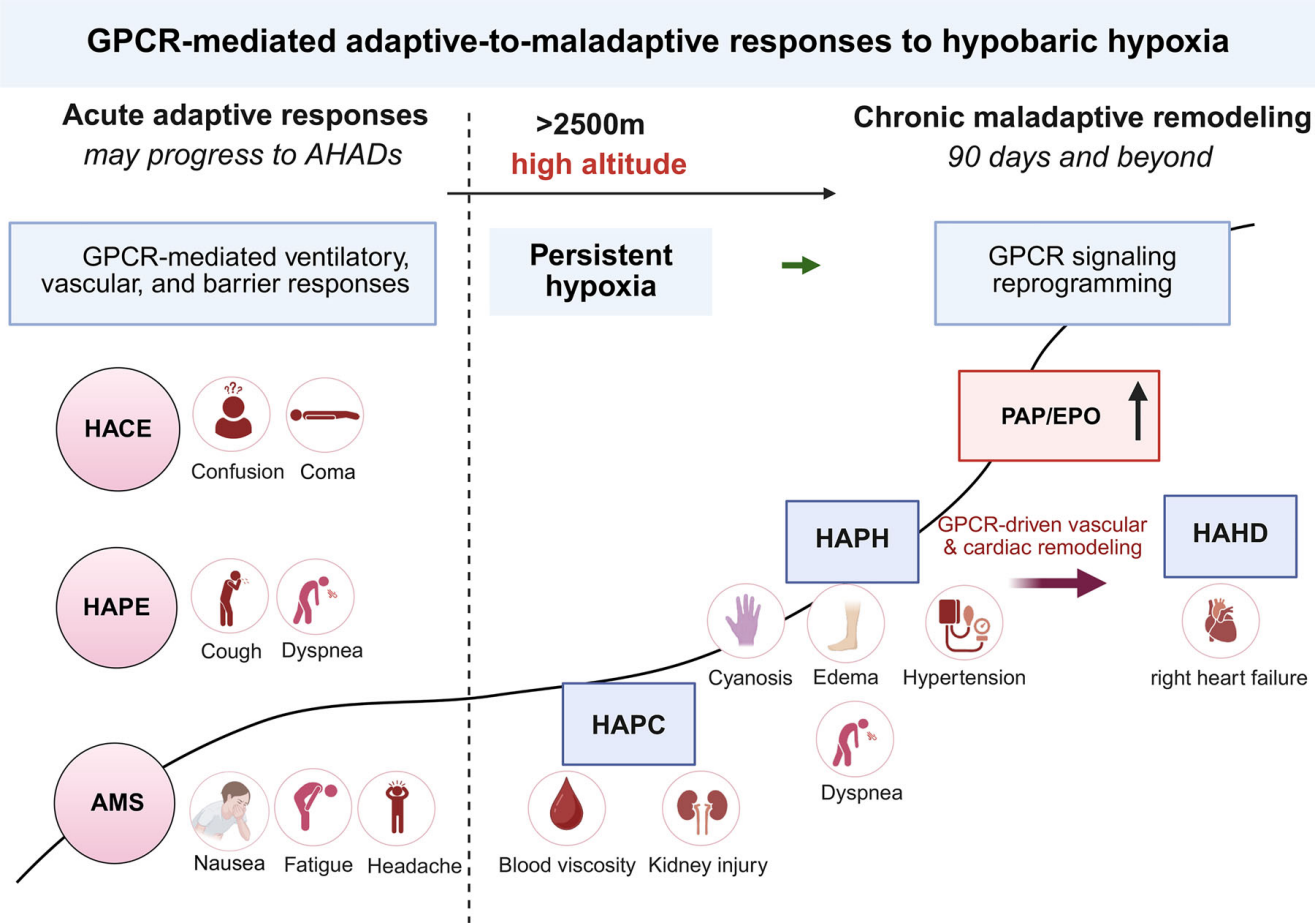
GPCR‐mediated continuum from adaptive to maladaptive responses to hypobaric hypoxia. This schematic illustrates how exposure to hypobaric hypoxia at high altitude (> 2500 m) elicits GPCR‐dependent responses that evolve from acute adaptation to chronic maladaptive remodeling. The left panel depicts acute GPCR‐mediated ventilatory, vascular, and barrier responses associated with acute high‐altitude diseases (AHADs), including acute mountain sickness (AMS), high‐altitude pulmonary edema (HAPE), and high‐altitude cerebral edema (HACE), with clinical manifestations such as fatigue, headache, cough, dyspnea, and cognitive disturbance. As hypoxia persists, a critical transitional state emerges, characterized by elevated pulmonary arterial pressure (PAP) and erythropoietin (EPO) levels. The right panel shows long‐term exposure (≥ 90 days) leading to GPCR signaling reprogramming and GPCR‐driven vascular and cardiac remodeling, culminating in high‐altitude pulmonary hypertension (HAPH), high‐altitude polycythemia (HAPC), and high‐altitude heart disease (HAHD), accompanied by cyanosis, edema, systemic hypertension, and right‐heart failure. Collectively, the figure emphasizes the central role of GPCR signaling in shaping the adaptive‐to‐maladaptive trajectory of hypobaric hypoxia at high altitude.

GPCRs represent the largest superfamily of cell surface membrane receptors, encoded by approximately 1000 genes [15, 16], including about 400 olfactory receptors [17]. They regulate a multitude of pivotal biological functions within the human body, including sensory perception, neurotransmission, and endocrine processes [15, 18]. Under hypoxic stress, GPCRs regulate multiple physiological systems, ranging from the activation of hypoxia‐inducible factor 1‐alpha (HIF‐1α) signaling to the modulation of cardiovascular and central nervous system functions [19]. In the cardiovascular system, GPCRs are involved in the regulation of blood pressure, heart rate, and myocardial contractility [20]. Additionally, GPCRs also modulate the release of vasoactive substances [21]. In the central nervous system, hypoxia directly affects the expression of GPCRs and the release of neurotransmitters, many of which act as GPCR ligands [22]. Prolonged hypoxia can induce receptor dysfunction, contributing to neurological disorders [23]. Emerging evidence indicates that GPCRs can influence mitochondrial function, thereby affecting the metabolic state and energy production of cells under hypoxic conditions [24]. This represents one of the pivotal mechanisms contributing to the development of HADs, such as pulmonary arterial hypertension.

High‐altitude exposure involves not only chronic hypobaric hypoxia but also additional environmental stressors, particularly cold exposure. Cold stress induces peripheral vasoconstriction and heat production, whereas hypoxic stress promotes hyperventilation, hemoconcentration, and erythropoietic responses to maintain oxygen delivery to tissues [25]. Cold weather has also been recognized as a risk factor that may modify susceptibility to high‐altitude illness [26]. Mechanistically, cold exposure activates sympathetic outflow and catecholamine release, thereby engaging adrenergic GPCR signaling to regulate vascular tone, cardiac workload, thermogenesis, and ventilatory control. Under hypoxic conditions, this adrenergic activation may interact with endothelin, adenosine, prostaglandin, and purinergic GPCR pathways to influence pulmonary vasoconstriction, endothelial permeability, inflammation, and metabolic adaptation. These combined effects may aggravate HAPE susceptibility by increasing pulmonary arterial pressure (PAP) and may also affect cerebral perfusion in AMS and HACE. During chronic exposure, repeated cold stress may further enhance vascular remodeling, right ventricular workload, and inflammatory signaling, thereby contributing to HAPH and HAHD. Experimental evidence that β_2_‐adrenergic receptor signaling modulates hypoxic pulmonary vasoconstriction (HPV) further supports the role of GPCRs as molecular nodes linking cold and hypoxia to high‐altitude adaptation and maladaptation [27].

Thus, GPCRs are central mediators of physiological responses to hypoxia and are increasingly recognized as key contributors to the development, progression, and potential treatment of altitude‐related disorders [28, 29]. In this review, we synthesize current knowledge on GPCR‐mediated mechanisms in hypoxic adaptation and maladaptation, highlight their roles in the pathogenesis of HADs, and explore opportunities for precision therapies targeting GPCRs in high‐altitude populations.

## 2 Acute High‐Altitude Diseases: From Clinical Features to GPCR Signaling

In recent years, with the rapid growth of tourism and economic development in high‐altitude regions, human activity has intensified, leading to a noticeable increase in the incidence of AHADs [30]. Among them, AMS, the most common AHAD, is characterized primarily by headache, along with nausea, dizziness, and fatigue (Table 1). These symptoms can appear as quickly as 6 h after arriving at the plateau, and usually do not last more than 24 h [31, 32]. The risk of AMS depends on individual susceptibility, age, health status, and prior high‐altitude experience, as well as environmental factors such as altitude, ascent rate, and weather conditions [30, 33, 34]. HACE and HAPE are much rarer than AMS. In a study of 14,050 individuals traveling to altitudes between 3500 and 5000 m, the overall incidence of HACE, HAPE, and AMS was 0.28%, 0.49%, and 51%, respectively [35] (Table 1).

**TABLE 1:** Prevalence and symptoms of AHADs.

Disease type	Prevalence	Symptom	Refs.
AMS	25%–40% at 3000–3500 m; 40%–90% at 4500–6000 m	Headache (primary symptom), nausea, dizziness, fatigue	[1, 31]
HACE	0.5%–1% at altitudes of 4000–5000 m; Rarely at 2500 m	Confusion, altered mental status, disorientation, decreased level of consciousness, focal neurological signs, and can evolve into a coma	[1, 36]
HAPE	1.7% at altitudes > 4000 m; some instances at 2500 m	Dyspnea with simple activities or at rest, cyanosis, cough with pink frothy sputum, respiratory distress	[1, 31, 37]

Abbreviations: AMS, acute mountain sickness; HACE, high‐altitude cerebral edema; HAPE, high‐altitude pulmonary edema.

The spectrum of AHADs ranges from the mild and self‐limiting symptoms of AMS to the life‐threatening complications of HACE and HAPE. We provide an overview of their core clinical features and relative symptom distributions (Figure 1). The pathological core of AMS is hypoxia‐related cerebral edema, which is closely associated with blood–brain barrier (BBB) disruption and glial responses, and directly contributes to clinical symptoms such as headache and nausea [38, 39, 40, 41, 42, 43, 44, 45, 46]. Thus, in severe cases, AMS can progress to HACE, a life‐threatening condition [47, 48], characterized by elevated intracranial pressure, impaired regulation of cerebral blood flow (CBF), and vasogenic edema [49]. HAPE primarily affects the lungs and is characterized by dyspnea, cough, and hypoxemia [50]. Its pathogenesis links a blunted hypoxic ventilatory response (HVR) [51, 52, 53] with endothelial dysfunction [50] and increased vascular permeability [53], leading to alveolar flooding.

Collectively, although AMS, HACE, and HAPE affect different organ systems, they share common hypoxic origins and overlapping pathogenic mechanisms. Central to their development are three inter‐related processes: (i) alterations in HVRs and pulmonary vascular reactivity [51, 52, 53], which determine systemic oxygen delivery and PAP; (ii) endothelial barrier dysfunction in the brain and lungs [38, 39, 40, 41, 44, 45, 46, 50, 53], leading to vasogenic cerebral edema or alveolar flooding; and (iii) disturbances in cerebrovascular and pulmonary hemodynamics [47, 50], which amplify tissue hypoxia and contribute to life‐threatening complications.

However, due to interspecies differences in high‐altitude adaptation, there remains a translational gap between animal models and human clinical conditions, leaving the molecular pathogenesis of AMS, HACE, and HAPE incompletely understood. Importantly, part of this gap arises from species‐specific and population‐specific genetic adaptations to hypoxia. Population‐based genomic studies have identified EPAS1 [54, 55] and EGLN1 [54, 56] as core high‐altitude adaptation genes in Tibetan and Andean populations, with strong signatures of positive selection. In addition to these evolutionarily selected genes, other regulators such as PPARA [54] and NOS3 [57, 58, 59, 60] contribute to metabolic and vascular adaptation under hypoxic conditions. Functionally, EPAS1, encoding HIF‐2α, and EGLN1, encoding prolyl hydroxylase 2, form a key oxygen‐sensing module that regulates HIF stability and hypoxia‐responsive transcription. Adaptive variants in these genes, especially in Tibetan high‐altitude populations, are associated with attenuated erythropoietic responses and lower hemoglobin concentrations, which may help reduce the risk of excessive erythrocytosis during chronic hypoxic exposure. In parallel, PPARA promotes fatty acid oxidation and optimizes energy utilization under hypoxia [54], whereas NOS3, encoding endothelial nitric oxide (NO) synthase, regulates NO production and vascular tone, contributing to blood flow regulation and susceptibility to HADs [57, 58, 59, 60]. In addition to HIF signaling, other canonical hypoxia‐responsive pathways, such as heat shock proteins (HSPs), further support cellular adaptation by preserving protein stability and limiting hypoxia‐induced stress [61]. Importantly, these genetic and molecular adaptations intersect with GPCR‐mediated signaling at multiple levels. For example, NO signaling downstream of NOS3 interacts with GPCR‐regulated vascular tone [62, 63], while HIF‐dependent transcriptional programs modulate the expression and responsiveness of multiple GPCR pathways, including adenosine [64] and endothelin signaling [65]. Taken together, these findings suggest that genetic adaptation provides an upstream regulatory framework that shapes GPCR‐mediated responses to hypoxia. Within this context, accumulating evidence has revealed the central role of GPCR signaling in these processes. The following sections summarize current knowledge of these mechanisms, with an emphasis on GPCR‐mediated signaling pathways that link hypoxic stress to maladaptive outcomes.

Here, we summarize current advances in GPCR‐mediated mechanisms in AHADs (Table 2) and highlight their implications for disease progression and therapeutic targeting. We provide an integrated overview of hypoxia‐induced pathophysiological responses leading to AMS, HACE, and HAPE, emphasizing the roles of pulmonary vasoconstriction, cerebrovascular dysregulation, and carotid body activation (Figure 2). Notably, GPCR subtypes such as adenosine, dopamine, serotonin, and purinergic receptors emerge as critical modulators within these pathways (Figure 2).

**TABLE 2:** Acute high‐altitude diseases targets.

Disease type	GPCRs	Effect and mechanisms	In vitro/in vivo/clinical trial	GPCR‐ligands	Refs.
AMS	D_2_R	D_2_R activation can inhibit HVR, and its antagonists (such as metoclopramide) alleviate headache symptoms in AMS.	Randomized, double‐blind, controlled trial (NCT01522326)	Agonist: Dopamine Antagonist: Metoclopramide	[66, 67, 68, 69, 70, 71, 72]
	Olfr78	Olfr78, as a hypoxia receptor, activates the activity of the carotid body.	Animal	Lactic acid	[73, 74]
	A_2_AR	Modulates hypoxic ventilatory and cerebrovascular responses through carotid‐body and cerebral vascular adenosine signaling.	Animal and human physiological studies	Antagonist: Caffeine, Selective agonist: CGS‐21680	[75, 76, 77, 78]
	5‐HT_1_B/_1_D	5‐HT_1_B/_1_D receptor agonism with sumatriptan has been clinically evaluated for AMS prophylaxis.	Prospective, double‐blinded, randomized, field‐based trial (ISRCTN87201238)	Agonist: Triptans	[79]
HACE	S1PR_1_	The activation of S1PR_1_ can maintain the integrity of BBB and prevent edema.	Not verified in HACE animal model, vitro trial	Selective agonist: SEW2871, CYM5442, A971432, FTY720	[80]
	S1PR_4_	Endothelial S1PR_4_ stabilizes the BBB; its deficiency increases vascular leak.	Animal	Agonist: S1P	[81]
	A_2_AR	Context‐dependent modulation of neuroinflammation and BBB permeability under hypoxia.	Animal	Antagonist: Caffeine, Agonist: Regadenoson, Selective agonist: CGS‐21680	[82, 83]
	A_2_BR	Suppresses hypoxia‐induced vascular leakage; maintains endothelial barrier integrity.	Animal	Selective antagonist: PSB1115 Agonist: BAY60‐6583	[84]
	TXA_2_R	The activation of TXA_2_R will aggravate the damage of the blood–brain barrier caused by hypoxia and affect cerebrovascular function.	It has not yet been verified in animal experiments	Agonist: U‐46619 Selective antagonist: S‐18886	[85, 86, 87]
HAPE	ETR	The activation of ET‐R can lead to an increase in pulmonary artery pressure.	No clinical progress	Agonist:ET‐1 Nonselective antagonists: Bosentan Selective antagonist: Ambrisentan	[11, 88]
	AT_1_R	Promotes hypoxic pulmonary vasoconstriction; AGTR1 variants may amplify HPV and increase HAPE susceptibility.	Genetic association study	NA	[89, 90]
	ADRB2	ADRB2 agonists can reduce the incidence of pulmonary edema in high‐altitude areas.	Double‐blind, randomized, placebo‐controlled study	Agonist: Salmeterol	[91, 92, 93]

Abbreviations: 5‐HT1B/1D, 5‐hydroxytryptamine 1B/1D receptor; A2AR, adenosine A2A receptor; A2BR, adenosine A2B receptor; ADRB2, β2‐adrenergic receptor; AT1R, angiotensin II Type 1 receptor; BBB, blood–brain barrier; ET‐1, endothelin‐1; ETR, endothelin receptor; GPCRs, G protein‐coupled receptors; HPV, hypoxic pulmonary vasoconstriction; HVR, hypoxic ventilatory response; ISRCTN, International Standard Randomised Controlled Trial Number; NCT, National Clinical Trial; Olfr78, olfactory receptor 78; S1P, sphingosine‐1‐phosphate; S1PR1, sphingosine‐1‐phosphate receptor 1; S1PR4, sphingosine‐1‐phosphate receptor 4; TXA2R, thromboxane A2 receptor.

**FIGURE 2:**
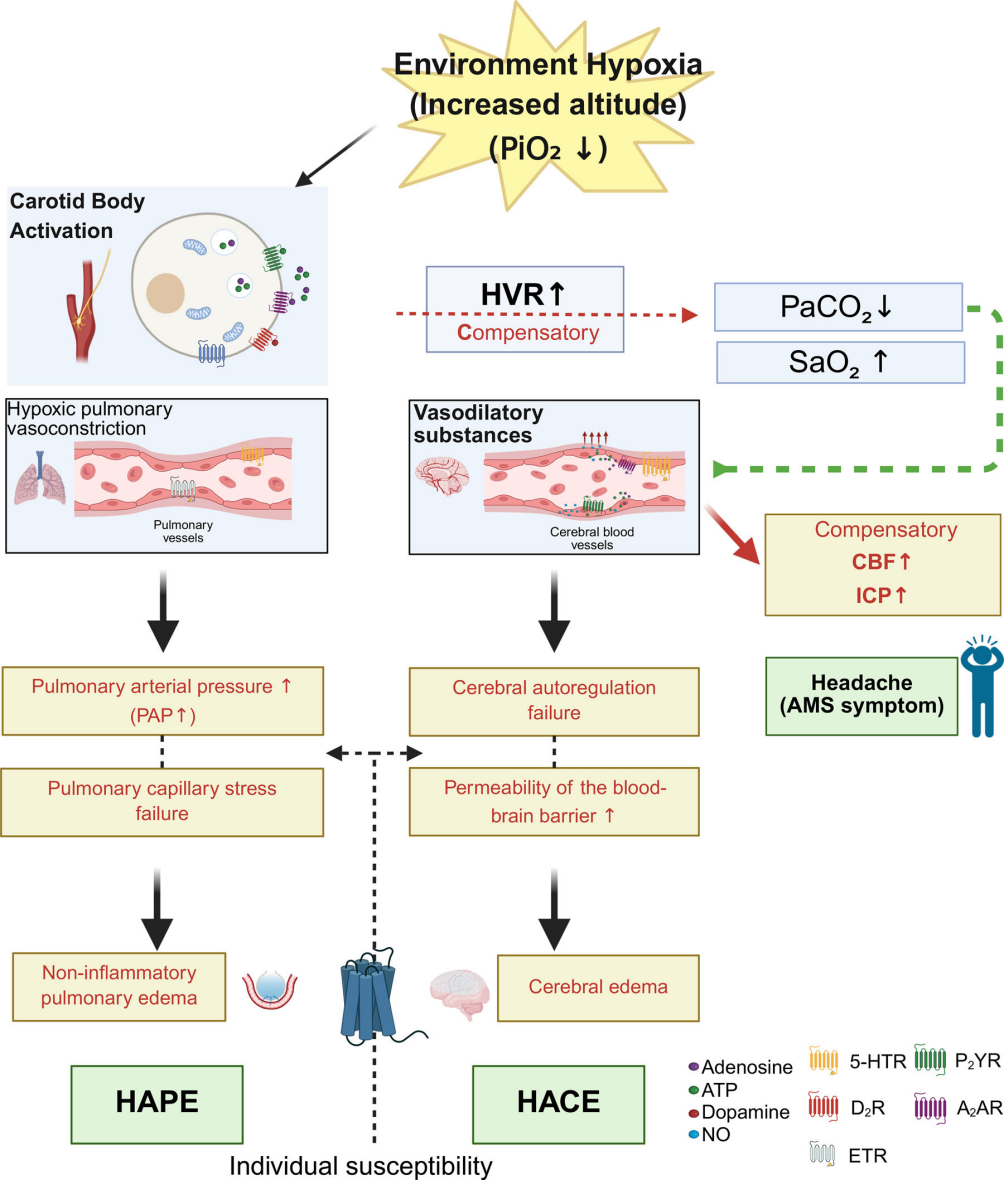
GPCR‐mediated mechanisms linking acute high‐altitude hypoxia to AMS, HAPE, and HACE. Acute high‐altitude hypoxia, caused by reduced inspired oxygen pressure (PiO_2_), activates carotid body chemosensing and increases the hypoxic ventilatory response (HVR). This compensatory response lowers PaCO_2_ and partially improves SaO_2_, but may also increase cerebral blood flow (CBF) and intracranial pressure (ICP), contributing to headache and AMS. In the pulmonary circulation, hypoxia‐induced GPCR signaling promotes hypoxic pulmonary vasoconstriction, elevated PAP, capillary stress failure, and noninflammatory pulmonary edema, leading to HAPE. In the cerebral circulation, vasodilatory mediators and impaired cerebrovascular regulation increase blood–brain barrier (BBB) permeability and cerebral edema, contributing to HACE. Key GPCR‐related pathways include adenosine, purinergic, dopamine, serotonin, endothelin, thromboxane, and adrenergic signaling.

### 2.1 GPCR‐Mediated Regulation of Ventilatory and Pulmonary Vascular Responses to Hypoxia

Individual susceptibility to AHADs is closely linked to two interconnected hypoxia‐driven physiological responses: the HVR and HPV [94]. Notably, individual susceptibility to AMS isstrongly associated with a diminished HVR [43, 95] and elevated metabolic demand [96], with genetic variability in the sensitivity of peripheral chemoreceptors to hypoxia further modulating the risk [52, 95]. Ventilatory regulation involves both peripheral chemoreceptors (carotid bodies) and the central nervous system [66].

In the carotid body, hypoxia activates Type I glomus cells, triggering the release of adenosine, adenosine triphosphate (ATP), dopamine, and NO [97] (Figure 2). These mediators regulate HVR through multiple GPCR pathways. Adenosine receptors (ARs), including the A_2_A receptor (A_2_AR) and A_2_B receptor (A_2_BR), and purinergic P2Y receptors (P2YRs) enhance excitability of afferent fibers and stabilize intracellular Ca^2^^+^, thereby reinforcing hypoxic ventilatory drive [75, 76, 98, 99, 100, 101, 102]. Likewise, dopaminergic signaling via dopamine D_2_ receptors (D_2_R) provides inhibitory feedback, attenuating neurotransmitter release and preventing excessive ventilatory excitation [66, 67, 68, 69, 70, 71].

Clinically, this dual regulation is reflected in pharmacological studies. Exogenous dopamine infusion suppresses carotid body activity and HVR by enhancing D_2_R‐mediated inhibition. This effect, however, is largely of experimental significance, since lowering ventilatory drive under hypoxia can worsen systemic oxygenation [68, 103]. D_2_R antagonists, like metoclopramide, enhance HVR and have been shown to reduce AMS‐related headache and nausea in field studies [72]. Thus, purinergic and dopaminergic signaling establish a bidirectional GPCR‐mediated regulation of HVR, balancing excitation and inhibition, with clinical implications for both experimental models and therapeutic strategies. Beyond their role in carotid body chemosensing, adenosine and purinergic receptors also participate in cerebrovascular regulation [104].

Beyond peripheral mechanisms, GPCRs in the central nervous system also modulate HVR. Activation of α‐adrenergic receptors (α‐ARs) and the β_2_‐adrenergic receptor (β_2_AR, encoded by ADRB2) in brainstem respiratory centers contributes to ventilatory facilitation by enhancing sympathetic outflow [105, 106]. Notably, the lactate‐sensitive olfactory receptor Olfr78 (mouse ortholog of human OR51E2) has been proposed as an auxiliary oxygen sensor [73, 74]. However, conflicting evidence suggests its role may be limited to fine‐tuning respiratory patterning rather than mediating core HVR, and thus its contribution to altitude adaptation remains uncertain.

Acute hypoxia elicits HPV, a compensatory mechanism to optimize ventilation‐perfusion matching [107]. A major characteristic of subjects with a predisposition to HAPE is their exaggerated HPV. Compared with HAPE‐resistant subjects, HAPE‐susceptible subjects have abnormally increased PAP when exposed to transient or prolonged hypoxia [94]. Moreover, excessive HPV can exacerbate PAP, elevate pulmonary vascular resistance, and promote the transudation of protein‐rich fluid into alveolar and interstitial spaces, leading to edema formation [108, 109, 110, 111]. GPCR signaling plays a pivotal role in this process: endothelin‐1 (ET‐1) activates endothelin receptors (ETRs), particularly the ETA receptor (ETAR), via the Gq/PLC pathway, driving vasoconstriction [112, 113]. ET receptor antagonists (ERAs), such as the ETA/ETB dual antagonist Bosentan, attenuate hypoxic pulmonary vascular hyper‐responsiveness and edema in experimental models and selected populations, though their clinical application in HAPE prevention remains under investigation [11, 88].

Beyond exaggerated HPV, additional GPCR‐mediated mechanisms contribute to HAPE pathophysiology. RAAS–AT_1_R signaling enhances vasoconstriction and sympathetic activity, and AGTR1 polymorphisms have been associated with increased HAPE risk [89, 114, 115, 116, 117]. Since these variants can elevate AT_1_R expression in other cardiovascular settings [118], it is plausible that they promote exaggerated HPV during hypoxia.

Together, impaired ventilatory drive and exaggerated pulmonary vascular responses disrupt oxygen homeostasis under hypoxia. This convergence indicates that GPCR‐mediated regulation of HVR and HPV plays a critical role in shaping individual susceptibility to altitude illness. Although initially adaptive, maladapted HVR limits systemic oxygenation, whereas exaggerated HPV elevates PAP and mechanical stress.

Maladaptation of HVR and HPV leads to characteristic changes in cerebrovascular and pulmonary hemodynamics, providing the physiological basis for subsequent vascular and epithelial injury.

### 2.2 GPCR‐Mediated Cerebrovascular and Pulmonary Hemodynamics

Abnormal hemodynamic patterns, including sustained vasoconstriction, elevated shear stress, and dysregulated CBF, emerge as defining consequences of impaired HVR and HPV. These hemodynamic disturbances exert profound effects on vascular stability and tissue oxygenation.

Hypoxia profoundly influences CBF and vascular tone. Under physiological conditions, cerebral autoregulation (CA) maintains relatively stable CBF across fluctuations in perfusion pressure. Hypoxia‐induced alterations in cerebrovascular and pulmonary hemodynamics represent a critical intermediate stage in the progression of altitude‐related pathology. Usually, acute hypoxia—within minutes of exposure—can transiently impair CA, leading to cerebral hypoperfusion and elevated intracranial pressure, which may contribute to AMS symptoms [6, 119, 120].

Although CA impairment has been proposed to correlate with AMS severity, current evidence remains inconsistent, as studies show no significant difference in CA function between AMS‐susceptible and AMS‐resistant individuals. This transient dysregulation likely represents a physiological adjustment to hypoxia rather than a pathological deficit.

CA fundamentally relies on the myogenic response of cerebral resistance vessels, in which GPCR signaling via G_12_/G_13_‐RhoA is required for vessel constriction in response to pressure changes [121, 122, 123]. Thus, CA impairment under hypoxia may involve dysregulation of GPCR‐mediated vascular tone in addition to metabolic and endothelial mechanisms.

Beyond large‐vessel autoregulation, fine‐tuning of CBF occurs at the capillary level through pericyte‐mediated control of capillary tone. Recent work has revealed that brain capillary pericytes possess a rich repertoire of ion channels and GPCRs, including adrenergic, purinergic, serotonergic, dopaminergic, and prostaglandin receptors, forming an “ion channel and GPCR toolkit” that allows them to sense and respond to neural and metabolic cues. Through these pathways, pericytes participate in neurovascular coupling and CO_2_ reactivity, complementing classical autoregulatory mechanisms. Under hypoxic conditions, dysregulation of pericyte GPCR signaling—particularly involving adenosine, P2Y, and prostaglandin receptors—may disturb microvascular flow patterns and contribute to the transient cerebral hypoperfusion characteristic of AMS. Together, these findings indicate that GPCR‐mediated signaling operates at multiple levels of the cerebrovascular network—from large arteries to capillary pericytes—to fine‐tune CBF under hypoxic stress.

In animal models, A_2_AR signaling mediates hypoxia‐induced vasodilation and CBF elevation, whereas A_2_AR knockout (KO) markedly attenuates these responses [124, 125]. Clinically, A_2_AR antagonists such as aminophylline and caffeine reduce CBF elevation and relieve AMS symptoms [77]. However, randomized studies suggest that AR‐mediated vasodilation is not obligatory in humans under hypoxia [78], implying the involvement of compensatory GPCR pathways that collectively maintain CBF homeostasis. Collectively, these findings highlight serotonergic and dopaminergic GPCRs as central targets for symptomatic AMS management. As discussed above, CBF is maintained through multiple regulatory routes, each engaging a wide spectrum of GPCRs that collectively orchestrate cerebrovascular adaptation to hypoxia. Although the precise number and hierarchy of GPCR pathways participating in this process remain unclear, elucidating their interactions will be essential for understanding the integrated control of cerebral perfusion under hypoxic stress.

In addition, 5‐HT_1_B/_1_D receptor agonists such as sumatriptan have been evaluated in a randomized double‐blind placebo‐controlled trial for AMS prevention (ISRCTN87201238), showing reduced AMS incidence in prophylactic use [79]. Similarly, the D_2_R antagonist metoclopramide was evaluated in a prospective double‐blinded randomized field‐based trial comparing metoclopramide with ibuprofen for high‐altitude headache and AMS treatment (NCT01522326) [72], suggesting potential symptomatic benefit, particularly in patients with nausea. Therefore, these observations highlight serotonergic and dopaminergic GPCRs as clinically explored targets for symptomatic management of AMS, while also underscoring the need for larger placebo‐controlled studies to define their therapeutic value.

As maladapted HVR and exaggerated HPV persist, the resulting sustained vasoconstriction and elevated shear stress impose chronic hemodynamic stress on the cerebral and pulmonary microvasculature. This hemodynamic burden, together with hypoxia‐induced inflammatory signaling, compromises vascular resilience and predisposes both endothelial and epithelial barriers to structural failure [126, 127].

### 2.3 GPCR‐Mediated Regulation of Endothelial and Epithelial Barrier Integrity

Under hypoxic stress, vascular and epithelial barriers, particularly the BBB, pulmonary endothelium, and alveolar epithelium, act as critical checkpoints maintaining fluid balance, tissue oxygenation, and organ homeostasis. Once disrupted, these barriers give rise to hallmark complications of AHADs, including vasogenic cerebral edema in HACE and alveolar flooding in HAPE. In the lung, combined endothelial and epithelial barrier dysfunction jointly increases vascular leakage, impairs alveolar fluid clearance, and promotes pulmonary edema formation. The endothelial and epithelial barriers constitute the two essential components of the blood–gas barrier. Damage to the epithelial layer impairs alveolar fluid clearance and further increases permeability, a characteristic feature of the progressive stage of HAPE [128].

Among GPCR‐mediated mechanisms, sphingosine‐1‐phosphate (S1P) signaling emerges as a pivotal regulator of BBB permeability. S1P influences endothelial barrier function, smooth‐muscle contraction, and vascular remodeling by activating its receptors S1PR_1_–S1PR_5_ [89, 129, 130]. Both mouse and human brain microvascular endothelial cells (BMECs) express S1PR_1_ and S1PR_2_. Chronic hypoxia downregulates S1PR_1_ in cerebrovascular endothelium, which is closely associated with BBB dysfunction. Notably, selective activation of S1PR_1_ with SEW2871 significantly alleviates hypoxia‐induced BBB impairment in animal models, underscoring its protective role [80]. In addition, S1PR_4_ is highly enriched in endothelial cells of the BBB, as shown by transcriptomic datasets. Functional studies demonstrate that S1PR_4_ KO or siRNA knockdown causes barrier disruption, supporting its role as a potential therapeutic target [81].

Beyond its vascular effects, A_2_AR signaling has been implicated in endogenous tissue‐protective and anti‐inflammatory responses to hypoxia in peripheral organs [131], indicating a broader role for adenosine signaling in hypoxic tissue adaptation. In the central nervous system, however, the effects of A_2_AR signaling on neuroinflammation and BBB integrity appear to be context dependent. Notably, the FDA‐approved A_2_AR agonist regadenoson (also known as Lexiscan) enhances permeability of the human BBB in vitro, facilitating immune and drug trafficking across the barrier [83]. Interestingly, mice lacking A_2_B ARs exhibit markedly increased vascular permeability across multiple tissues under hypoxic conditions, and endothelial A_2_BR plays a critical protective role in limiting hypoxia‐induced vascular leakage [84]. Thus, adenosine signaling exerts multitiered vascular protection under hypoxia, with A_2_AR dynamically modulating BBB permeability and neuroinflammatory responses, whereas A_2_BR contributes more consistently to endothelial barrier protection under hypoxia.

Additionally, in HACE, vascular dysregulation is further amplified by thromboxane A_2_ (TXA_2_) signaling via the TXA_2_ receptor (TXA_2_R), which promotes vasoconstriction, platelet aggregation, and microthrombus formation, aggravating BBB leakage and impairing CBF [85, 86, 87]. TXA_2_ signaling may contribute to hypoxia‐associated cerebrovascular dysregulation through its vasoconstrictive and proaggregatory effects; however, direct evidence establishing TXA_2_R as a causal therapeutic target in HACE remains limited [86].

In the lung, GPCR‐dependent mechanisms also shape barrier function. Impaired ADRB2 signaling decreases epithelial sodium channel (ENaC) activity, thereby reducing alveolar fluid clearance and predisposing the lung to edema accumulation. ADRB2 agonists such as salmeterol counteract this defect and attenuate HAPE‐like pulmonary edema in experimental and clinical settings [91, 92, 93].

Taken together, these findings show that multiple GPCR pathways—including S1PR_S_, A_2_AR, TXA_2_R, and ADRB2—play essential roles in maintaining endothelial and epithelial barrier stability under hypoxic stress. Their combined actions help determine whether hypoxia results in barrier preservation or breakdown, thereby shaping susceptibility to HACE and HAPE. Barrier failure does not occur in isolation but arises alongside profound alterations in cerebrovascular and pulmonary hemodynamics, which are likewise modulated by GPCR signaling.

Collectively, the GPCR‐dependent ventilatory, hemodynamic, and barrier responses discussed above define the acute phase of high‐altitude adaptation and injury. However, when hypoxia becomes sustained, these same pathways undergo long‐term remodeling that fundamentally alters cerebrovascular and cardiopulmonary function. This transition from acute compensation to chronic maladaptation forms the basis of CHADs.

## 3 Chronic High‐Altitude Diseases: From Clinical Features to GPCR Signaling

Prolonged residence at high altitude triggers complex structural and molecular remodeling across multiple organ systems. Chronic hypoxia sustains erythropoietin (EPO) production and activates hypoxia‐responsive GPCR pathways that regulate erythropoiesis, vascular tone, and metabolic adaptation. While these mechanisms initially enhance oxygen transport and tissue perfusion, their persistent activation can result in pathological remodeling—manifesting as excessive erythrocytosis, pulmonary hypertension (PH), and right‐heart overload. Such transitions from adaptive to maladaptive states underscore the dual role of GPCR signaling in high‐altitude physiology: promoting short‐term acclimatization yet predisposing to chronic disease when hypoxic stress becomes sustained.

CHADs represent maladaptive responses to sustained hypobaric hypoxia and are most commonly manifested as HAPC, HAPH, and HAHD [6, 7, 8]. CMS (Monge disease) represents a related chronic hypoxia syndrome characterized primarily by excessive erythrocytosis and associated clinical manifestations, their prevalence varies by geography, ethnicity, and altitude [6]. On the Qinghai–Tibet Plateau, prevalence rises from 1.05% at 2267–2980 m to 5.83% at 4006–5226 m [6].

Clinically, CHADs are characterized by fatigue, headache, sleep disturbances, and exertional dyspnea, with progression to cardiopulmonary compromise if untreated [6, 12] (Table 3). HAPC presents with symptomatic polycythemia and exacerbated hypoxemia [132, 133], HAPH with progressive pulmonary hypertension, and HAHD with right ventricular hypertrophy and dysfunction [134]. These syndromes substantially impair quality of life and may become life‐threatening.

**TABLE 3:** Prevalence and symptoms of CHADs.

Disease type	Prevalence	Symptom	Refs.
HAPC	5.0%–18.0% on the Qinghai–Tibetan Plateau, varies by altitude and ethnicity	Headache, fatigue, dizziness, sleep disturbance, and hyperviscosity‐related manifestations	[135, 136]
HAPH	5.0%–18% in high‐altitude populations at risk	Fatigue, shortness of breath, cognitive deficits, cough, and in advanced cases hepatosplenomegaly and overt right‐sided heart failure	[137]
HAHD	—	Exertional dyspnea, fatigue, exercise intolerance, and manifestations of right‐heart dysfunction	[8]

Abbreviations: HAHD, high‐altitude heart disease; HAPC, high‐altitude polycythemia; HAPH, high‐altitude pulmonary hypertension.

In this section, we review the latest insights into the pathophysiological mechanisms of CHADs (Table 4) and discuss their relevance to disease progression and potential therapeutic strategies. We present a comprehensive framework of the three interconnected pathological axes contributing to CHADs, focusing on hematologic adaptation (Figure 3), pulmonary vascular remodeling (Figure 4), and cardiac remodeling. Specifically, we highlight the role of excessive erythropoiesis, red blood cell metabolic reprogramming, elevated PAP, and chronic pressure overload as pivotal factors influencing these pathological processes.

**TABLE 4:** Chronic high‐altitude diseases targets.

Disease type	GPCR	Effect and mechanism	In vitro/in vivo/clinical trial	GPCR‐ligands	Refs.
HAPC	GPR30 (GPER1)	GPER1 interacts with HIF‐1α signaling under hypoxic conditions; its direct contribution to the HIF‐2α–EPO axis and HAPC remains unconfirmed.	In vivo	Agonist: Estrogen, G1	[138, 139]
	AT_1_R	The activation of AT_1_R in the kidneys and hematopoietic stem cells promotes the release of EPO.	In vitro	Agonist: Ang II	[140, 141, 142]
	A_2_BR	Activation of A_2_BR increases the oxygen delivery capacity of erythrocytes and promotes S1P release.	In vitro	Agonist: Adenosine	[143, 144, 145, 146, 147, 148]
HAPH	ETAR	The ETA receptor of smooth muscle promotes vasoconstriction and proliferation.	Clinical trial	Antagonist: Bosentan	[112, 149, 150]
	IP	IP mediates vasodilation, and prostacyclin analogues exert therapeutic effects on pulmonary hypertension through IP.	Animal and clinical	Agonist: PGI2, Epoprostenol, Treprostinil, Selexipag	[151, 152, 153, 154, 155]
	TXA_2_R (TP)	TXA_2_ mediates vasoconstriction and smooth‐muscle proliferation through TXA_2_R.	Animal	Agonist: U‐46619 Antagonists: Terutroban	[156, 157, 158]
	E receptor 3 (EP3)	Gαi‐coupled prostaglandin E receptor 3 (EP3) receptor counteracts vasodilatory pathway.	Animal	Agonist: Sulprostone Antagonist: L‐798106	[159]
	CXCR4	CXCR4 mediates the proliferation of pulmonary artery smooth muscle and aggravates vascular remodeling in the rat model of pulmonary hypertension.	Animal	Inhibitor: AMD3100 (Plerixafor)	[160, 161]
	5‐HT_2_B	The 5‐HT_2_B receptor promotes endothelial cell proliferation and also plays a key regulatory role in endothelial–smooth‐muscle interactions.	Animal	Partial agonist: VU6047534	[162, 163, 164, 165, 166]
	ETBR	Endothelial cell ETB receptors mediate vasodilation and ET‐1 clearance, while smooth‐muscle ETB receptors promote vasoconstriction and proliferation.	In vitro and human vascular studies	Antagonist: Bosentan	[167, 168, 169, 170]
	DP1	DP1 mediates vasodilation, and DP1 deficiency can lead to the aggravation of pulmonary hypertension in mouse models.	Animal	Agonist: PGD2, BW245C, AS702224,	[171]
	A_2_AR	The activation of adenosine A_2_A receptor can also promote vasodilation, thereby alleviating HAPH.	Animal	Selective agonist: CGS‐21680	[172, 173]
	APLNR	APLNR agonism reverses vascular remodeling in SuHx (Sugen5416/normobaric hypoxia) rat HAPH model.	Animal	G protein‐biased APLNR agonist MM07, WN561	[174, 175, 176, 177]
	S1PR_2_	The activation of S1PR_2_ in smooth‐muscle cells promotes the proliferation of smooth muscle and aggravates HAPH.	Animal	Antagonist: JTE‐013	[178, 179]
	S1PR_1_	Endothelial cell S1PR_1_ mediates blood flow and vasodilation caused by S1P.	Not in HAPH animal model	Selective agonist: SEW2871, CYM5442, A971432, FTY720	[180, 181, 182]
	GPR75	GPR75 inhibits the cAMP vasodilation signaling pathway and assists in hypoxia‐induced contraction and remodeling.	Animal	orphan GPCR	[183]
	GPR146	GPR146 induces pyroptosis of endothelial cells and proliferation of smooth muscle under hypoxic conditions.	Animal	Orphan GPCR	[184, 185]
HAHD	APLNR	The β‐arrestin signaling pathway mediated by APLNR. promotes cardiac remodeling.	Animal	G protein‐biased APLNR agonist: MM07, WN561	[175, 177, 186]
	GPR30 (GPER1)	Estrogen decreased HIF 1α and MIF expression in cardiomyocytes under hypoxic conditions through GPER agonist G1.	In vitro and animal	GPER agonist: Estrogen, G1	[187]

Abbreviations: 5‐HT_2_B, 5‐hydroxytryptamine 2B receptor; APLNR, apelin receptor; cAMP, cyclic adenosine monophosphate; CXCR4, C‐X‐C motif chemokine receptor 4; DP1, prostaglandin D2 receptor 1; EP3, prostaglandin E receptor 3; EPO, erythropoietin; ET‐1, endothelin‐1; ETAR, endothelin A receptor; ETBR, endothelin B receptor; GPER1 (GPR30), G protein‐coupled estrogen receptor 1; GPR146, G protein‐coupled receptor 146; GPR75, G protein‐coupled receptor 75; HIF‐1α, hypoxia‐inducible factor‐1α; IP, prostacyclin receptor; MIF, macrophage migration inhibitory factor; PGD2, prostaglandin D2; PGI2, prostacyclin; SuHx, Sugen5416/normobaric hypoxia; TP, thromboxane receptor.

**FIGURE 3:**
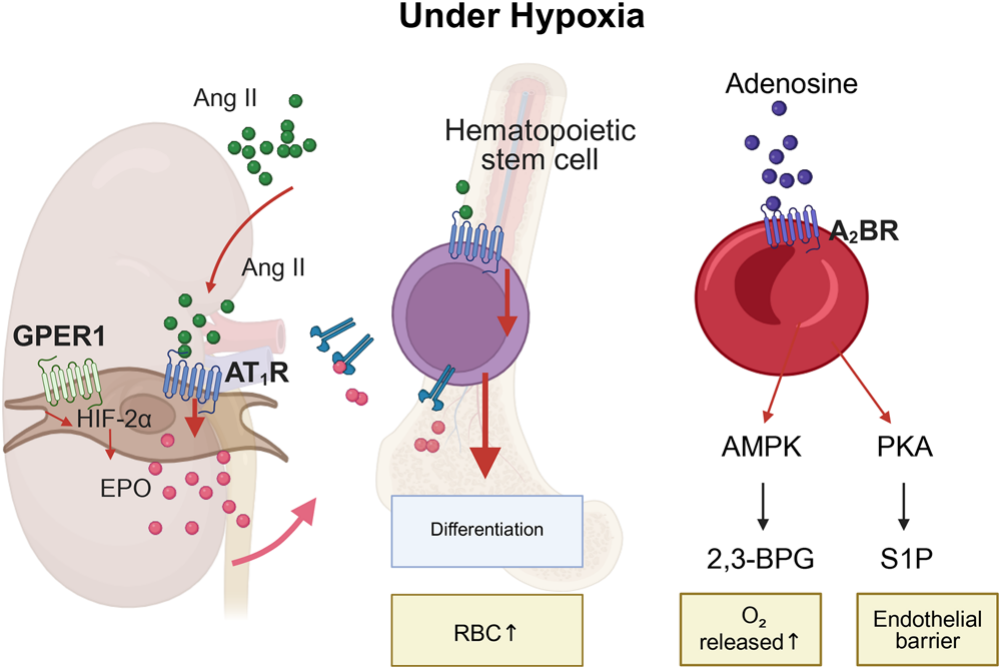
GPCR‐mediated regulation of erythropoiesis and erythrocyte adaptation under chronic high‐altitude hypoxia. Chronic hypobaric hypoxia activates renal hypoxia signaling and promotes erythropoietin (EPO) production through the hypoxia‐inducible factor 2α (HIF‐2α) pathway, with G protein‐coupled estrogen receptor 1 (GPER1) and angiotensin II (Ang II)–angiotensin II Type 1 receptor (AT_1_R) signaling potentially intersecting with HIF pathways, while AT1R signaling contributes more directly to EPO regulation. Increased EPO stimulates hematopoietic stem cell differentiation and erythrocyte production, resulting in elevated red blood cell (RBC) levels during chronic high‐altitude exposure. In mature erythrocytes, adenosine A_2_B receptor (A_2_BR) signaling enhances oxygen release and supports endothelial barrier protection, partly through metabolic mediators such as 2,3‐bisphosphoglycerate (2,3‐BPG) and sphingosine‐1‐phosphate (S1P). Together, these GPCR‐dependent pathways coordinate erythropoietic expansion, oxygen delivery, and vascular adaptation, but sustained activation may contribute to maladaptive erythrocytosis in chronic high‐altitude disease.

**FIGURE 4:**
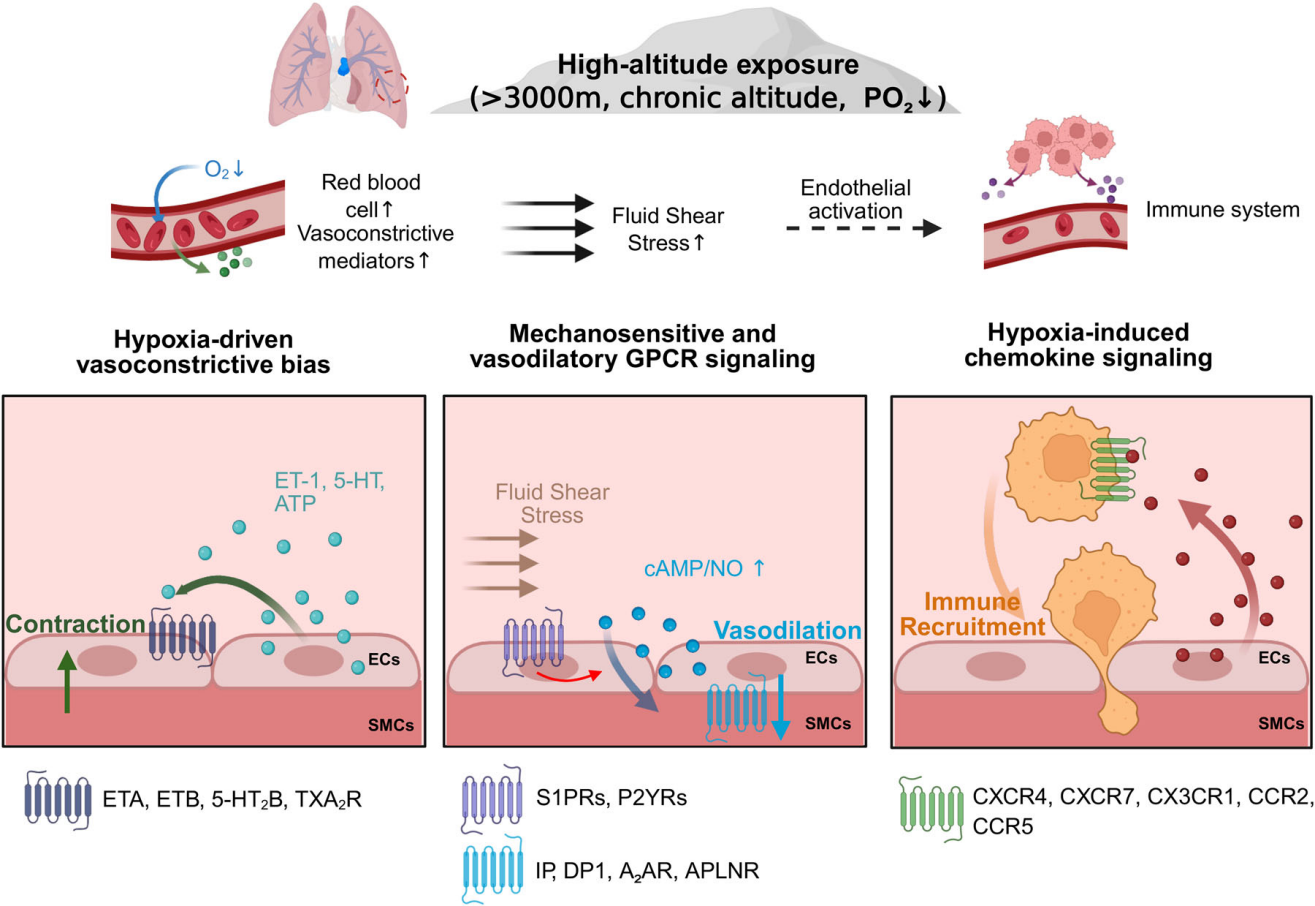
GPCR‐mediated pulmonary vascular responses to high‐altitude hypoxia. Chronic exposure to high altitude (> 3000 m) and sustained hypobaric hypoxia disrupt pulmonary vascular homeostasis and promotes HAPH. Hypoxia enhances the release of vasoconstrictive mediators such as endothelin‐1 (ET‐1), serotonin (5‐HT), and ATP, which activate GPCR signaling in pulmonary arterial smooth muscle cells (SMCs), increasing intracellular Ca^2^^+^ signaling and driving SMC contraction. In parallel, altered fluid shear stress activates endothelial mechanosensitive purinergic signaling, particularly P2Y_2_ receptor–dependent pathways, modulating cyclic adenosine monophosphate (cAMP)/nitric oxide (NO)‐mediated vasodilatory responses. Persistent hypoxia also promotes endothelial dysfunction and immune‐cell recruitment through chemokine–GPCR axes, such as C‐X‐C motif chemokine ligand 12 (CXCL12)–C‐X‐C motif chemokine receptor 4 (CXCR4), thereby amplifying vascular inflammation, endothelial injury, and pulmonary vascular remodeling. Together, the imbalance between vasoconstrictive, vasodilatory, mechanosensitive, and inflammatory GPCR pathways contribute to elevated PAP and progressive pulmonary vascular remodeling during chronic high‐altitude disease.

### 3.1 Erythropoiesis and Hematologic Adaptation

Notably, genetic adaptation mechanisms also contribute to chronic hypoxia responses. Key hypoxia adaptation genes such as EPAS1 and EGLN1, which have been extensively characterized in high‐altitude populations, play central roles in regulating the HIF‐2α–EPO axis and erythropoietic adaptation [54, 55, 56]. Excessive erythrocytosis in HAPC represents the hallmark of maladaptive hematologic adaptation to chronic hypoxia. Clinically, it is characterized by excessive erythrocyte proliferation, increased hemoglobin levels, elevated hematocrit, and reduced arterial oxygen saturation [119, 120, 188]. Its pathogenesis is centrally governed by HIF‐2α activation in peritubular fibroblasts, which stimulates EPO secretion under renal hypoxia [30, 114, 116, 136]. Elevated HIF‐2α and EPO expression in HAPC bone marrow correlates with hemoglobin levels [189], underscoring the centrality of the HIF‐2α–EPO axis.

Under hypoxia, the G protein‐coupled estrogen receptor 1 (GPER1, also known as GPR30), a membrane‐bound estrogen receptor, has been reported to interact with HIF signaling. Experimental studies in non‐HAPC cellular models suggest reciprocal crosstalk between GPER1 and HIF‐1α, with HIF‐1α promoting GPER1 expression and GPER1 signaling, in turn, modulating HIF‐1α stability and transcriptional activity [19, 138, 139]. However, direct evidence linking GPER1 to the HIF‐2α–EPO axis or excessive erythrocytosis in HAPC remains limited. Therefore, the potential contribution of GPER1 to sex‐related differences in HAPC susceptibility should currently be considered a mechanistically plausible but unconfirmed hypothesis.

Sex‐based epidemiological differences provide clinical context for GPER1‐related mechanisms in HADs. A meta‐analysis reported a modestly higher prevalence of AMS in women than in men, although substantial heterogeneity limits definitive interpretation [190]. By contrast, HAPE incidence may be lower in women, but the available evidence remains limited. For chronic mountain sickness (CMS), premenopausal women generally show a lower prevalence than men living at high altitude, consistent with evidence that estrogen can modulate erythropoiesis and protect against excessive erythrocytosis under chronic hypoxia [191]. These observations support the need to consider sex as a biological variable in GPCR‐mediated hypoxic adaptation and maladaptation.

Concurrently, the renin–angiotensin system amplifies erythropoiesis through the Ang II–AT_1_R pathway. In renal tissues, AT_1_R activation enhances EPO synthesis, while in the bone marrow, it potentiates JAK2–STAT5 signaling downstream of the EPO receptor, increasing erythroid proliferation [141, 142]. Additionally, AT_1_R may facilitate JAK2 activation via Gαq‐mediated PKC signaling or β‐arrestin–scaffolded pathways, thereby amplifying erythropoietic responses [140]. Together, these findings suggest that GPCR signaling intersects with hypoxia‐responsive and erythropoietic pathways at multiple levels. Whereas AT_1_R has more direct links to EPO production and erythropoiesis, the contribution of GPER1 to HAPC remains supported mainly by indirect evidence of crosstalk with HIF signaling.

Beyond the HIF–EPO cascade, adenosine signaling provides a second layer of regulation over erythropoiesis and erythrocyte function [192], such as the CD73–adenosine–AMPK axis. Hypoxia elevates extracellular adenosine through CD73‐mediated AMP dephosphorylation, which engages multiple ARs expressed on erythroid progenitors and mature red cells. At high concentrations, adenosine reduces erythroid proliferation largely via A_3_ receptor (A_3_R) activation, whereas impaired terminal maturation is mediated by both A_3_R and A_2_BR. Notably, selective A_2_BR agonism with BAY 60–6583 suppresses terminal erythroid differentiation in human CD34^+^ progenitors, highlighting its context‐dependent inhibitory role in erythropoiesis [193]. By contrast, A_3_R uniquely promotes myeloid skewing, further illustrating that adenosine orchestrates lineage decisions in hematopoietic progenitors.

In contrast, in mature erythrocytes, A_2_BR activation assumes an adaptive role, enhancing oxygen delivery and alleviating tissue and renal hypoxia, by stimulating AMP‐activated protein kinase (AMPK) and the PKA–/ERK1/2–SphK cascade. This coordinated signaling increases bisphosphoglycerate mutase activity, elevates 2,3‐bisphosphoglycerate (2,3‐BPG) levels, and promotes S1P production [143, 144, 145, 146, 148]. Consequently, the A_2_BR–AMPK–SphK1 axis functions as an adaptive pathway supporting oxygen transport and protecting against renal hypoxia under chronic low‐oxygen exposure [147]. In addition, circulating S1P also exerts vascular protective effects by maintaining endothelial barrier integrity and suppressing inflammation [130].

Notably, many of these mechanisms were first characterized in sickle cell disease, where chronic overactivation of the adenosine–A_2_BR–S1P pathway contributes to vaso‐occlusion and inflammation. In contrast, more moderate activation under conditions of high‐altitude adaptation is predominantly protective, highlighting the context‐ and stage‐dependent duality of GPCR signaling in hypoxic hematology.

Collectively, these mechanisms demonstrate that GPCRs regulate both EPO synthesis and erythroid sensitivity/metabolic reprogramming, thus reinforcing erythrocyte expansion in HAPC. Importantly, excessive erythrocytosis not only augments oxygen‐carrying capacity but also markedly increases blood viscosity, imposing additional hemodynamic load on the pulmonary circulation. This hyperviscosity, when combined with hypoxia‐induced vascular remodeling, creates a fertile ground for the development and progression of HAPH. Hence, hematologic maladaptation and pulmonary vascular pathology should be viewed as interconnected axes of chronic hypoxia‐related disease, rather than isolated processes, a concept further elaborated in the subsequent section on pulmonary vascular remodeling.

### 3.2 GPCR‐Mediated Pulmonary Vascular Remodeling and Hemodynamics

Hematologic maladaptation feeds directly into vascular signaling. Under sustained hypoxia and shear abnormalities, red blood cells are not inert carriers but dynamic mediators: they release ATP that activates endothelial P2Y_2_ receptors to alter NO bioavailability, export S1P that signals through S1PR_1_ to influence barrier integrity and tone, and convey NO–related species that further modulate vasoreactivity. Together with endothelin and chemokine axes, these inputs bias the pulmonary circulation toward GPCR‐dependent vasoconstriction, inflammation, and structural remodeling—bridging the erythrocytosis of HAPC to the hemodynamic and architectural changes of HAPH.

PH at high altitude reflects maladaptive remodeling of the pulmonary circulation under sustained hypoxia [8]. Hypoxia potentiates the release of vasoconstrictive mediators and activates immune‐inflammatory pathways, which collectively drive structural remodeling of the pulmonary vasculature [107, 161, 194]. This remodeling is characterized by vascular wall thickening, increased permeability, and disturbed vasoactive balance under sustained or intermittent hypoxia. GPCRs serve as central orchestrators of vasoconstrictive and remodeling processes. Vasoconstriction is primarily mediated through Gα_q/11_‐ and Gα_12/13_‐coupled GPCRs, which elevate intracellular Ca^2^^+^ and activate RhoA signaling, whereas Gαs‐coupled GPCRs mediate vasodilation via cAMP signaling [195, 196]. Hypoxia shifts this balance toward constriction, while inflammatory GPCRs such as C‐X‐C chemokine receptor type 4 (CXCR4) further exacerbate vascular remodeling [160]. In addition, hypoxia‐sensitive mechanosensitive GPCRs such as P2Y_2_R respond to shear stress by modulating NO release, while inflammatory GPCRs such as CXCR4 drive immune cell recruitment and vascular remodeling. As summarized in Figure 4, ET‐1/ETAR signaling mediates vasoconstriction, P2Y_2_R activation regulates NO release under fluid shear stress, and CXCL12–CXCR4 signaling facilitates inflammatory cell infiltration.

Collectively, hypoxia shifts the vasoactive balance toward constriction, amplifies mechanical stress responses, and promotes chronic inflammation, all converging to accelerate HAPH progression. These GPCR‐mediated pathways collectively shape the maladaptive pulmonary vascular response to hypoxia and represent potential targets for therapeutic intervention.

#### 3.2.1 Pulmonary Vasoconstriction

In HAPH, ET‐1 signaling plays a central role in the pathophysiology of PH [149, 197]. Hypoxia induces the upregulation of ET‐1 and its receptors, enhancing pulmonary vascular resistance [198, 199]. Endothelial ETB receptors facilitate vasodilation and clearance of circulating ET‐1 [167, 168, 169, 170], whereas smooth‐muscle‐expressed ETA/ETB mediate vasoconstriction and cellular proliferation. This mechanistic understanding is corroborated by clinical evidence demonstrating that dual ETA/ETB antagonism with bosentan achieves greater reductions in PAP compared to oxygen therapy alone [112, 149, 150]. In addition to altered ET‐1 signaling, genetic variation in ETRs may also contribute to interindividual variability in pulmonary vascular responses to hypoxia. Variants in EDNRA, encoding ETAR, have been linked to PH and may affect PAP regulation [200], whereas variation in EDNRB, encoding ETBR, has been associated primarily with high‐altitude adaptation rather than overt disease susceptibility [201]. These findings suggest that ETR genetics may modulate hypoxic pulmonary vascular responses, although their value for predicting HAPE or HAPH risk requires further validation.

Beyond the endothelin axis, several other vasoconstrictor pathways contribute to pulmonary hypertensive remodeling, including serotonergic, TXA_2_, and RAAS pathways. Elevated 5‐HT in PAH patients drives vascular remodeling via 5‐HT receptor activation [202, 203], particularly via the 5‐HT_2_B subtype [165, 166]. Murine models lacking 5‐HT_2_B receptors exhibit resistance to hypoxic PAH [166, 204], while selective antagonists attenuate hemodynamic and structural remodeling [162, 164]. However, the widespread central and peripheral distribution of 5‐HT receptors confers a risk of adverse effects—including migraine, fibrosis, and valvopathy [164, 205], prompting the development of novel peripherally restricted ligands to improve therapeutic safety [162, 163, 164]. Similarly, TXA_2_ exerts potent constrictive and proliferative effects via TXA_2_R [157, 158], further reinforcing the multiaxis nature of vasoconstrictive signaling in PH.

Together, these vasoconstrictor pathways establish a sustained high‐resistance pulmonary vascular state that drives the progression of HAPH. At the same time, a distinct set of vasodilatory GPCR pathways works to counterbalance excessive vasoconstriction, preserve perfusion, and limit hypoxia‐induced remodeling. The dynamic balance between these opposing forces ultimately shapes pulmonary vascular outcomes in HAPH.

#### 3.2.2 Pulmonary Vasodilatory GPCR Signaling

Given the dominance of vasoconstrictive signaling, therapeutic strategies for PH are increasingly focused on augmenting compensatory vasodilatory signaling. Prostacyclin receptor (IP; PTGIR) agonists raise cAMP levels, promote relaxation of pulmonary arterial smooth‐muscle cells (PASMCs) and improve clinical outcomes in PH [151, 152, 153, 154, 155]. IP deletion aggravates hypoxia‐induced PH in experimental models [151, 152], recapitulating key features of HAPH. Similarly, the prostaglandin D_2_ receptor 1 (DP1, also known as PTGDR) activated by prostaglandin D_2_ and coupled to Gαs, exerts protective effects. DP1 loss exacerbates PH, whereas agonism ameliorates vascular remodeling [171]. Conversely, increased expression of the Gαi‐coupled prostaglandin E receptor 3 (EP3) receptor counteracts vasodilatory pathways [153, 155]. Together, these findings highlight that prostaglandin receptors exert divergent effects in PH: IP and DP1 promote vasodilation and vascular protection, whereas EP3 counteracts these pathways to favor remodeling. Selective modulation of these receptor subtypes thus represents a promising therapeutic approach.

Activation of A_2_AR can also promote pulmonary vasodilation through a Gαs protein‐coupled mechanism, inducing vasodilation and mitigating HAPH pathology [172, 173]. A_2_AR KO models induce spontaneous vascular thickening, and A_2_AR agonists prevent remodeling under chronic hypoxia. Similarly, the apelin‐APLNR(APJ) axis is protective: hypoxia reduces APLNR despite upregulated apelin, indicating a receptor–ligand mismatch [174, 176]. This hypoxia‐induced dysregulation may contribute to pathological vascular remodeling and disease progression. Consistent with these findings, APLNR agonism reverses vascular remodeling in SuHx (Sugen5416/ normobaric hypoxia) rat HAPH models [176].

In contrast, vasodilatory GPCR pathways such as IP, DP1, A_2_AR, and APLNR promote cAMP‐dependent relaxation and confer vascular protection, while EP3 activation opposes these effects. Selective activation of protective GPCRs and inhibition of maladaptive ones may restore the vasodilatory–vasoconstrictive balance in HAPH.

#### 3.2.3 Mechanical Perception of Pulmonary Vessels

Chronic hypoxia disrupts protective vascular mechanosensation pathways, notably impairing shear stress–mediated NO release [206, 207]. S1P signaling bridges mechanical cues to vascular responses [179]. Elevated S1P levels have been reported in plasma and vascular tissues of PAH patients [178], and in preclinical hypoxic HAPH models [178]. Specific activation of S1PR_2_ promotes vasoconstriction and remodeling, inhibition of S1PR_2_ with JTE‐013 has been shown to attenuate HAPH severity [178, 179]. Conversely, endothelial S1PR_1_ activation promotes vascular barrier integrity and sustains NO bioavailability [180, 181, 182], suggesting divergent roles in vascular adaptation to hypoxia and in the pathogenesis of HAPH.

The purinergic signaling system introduces a further layer of complexity. P2Y_2_R expression is upregulated in hypoxic lungs [208], where it mediates ATP‐induced signaling that contributes to pulmonary vascular remodeling. This upregulation may represent an adaptive mechanism to sustain endothelial function under hypoxic stress; however, dysregulated purinergic signaling in PAH patient‐derived pulmonary artery endothelial cells suggests that altered P2Y signaling also contributes to endothelial dysfunction and vascular remodeling [209]. P2Y_2_R therefore likely functions as a mechanosensitive, NO‐coupled module, although its potential influence on smooth‐muscle proliferation remains to be clarified. In contrast, ADP‐driven activation of P2Y_1_R and P2Y_12_R induces vasoconstriction [210], underscoring the opposing roles of purinergic receptor subtypes in the regulation of pulmonary vascular tone.

#### 3.2.4 Immune–Inflammatory GPCR Mechanisms

In contrast to the well‐characterized molecular mechanisms of other forms of PH, the pathophysiological basis of HAPH remains to be fully elucidated. Nevertheless, hypoxia is a potent driver of inflammatory cell recruitment and proremodeling inflammatory responses within the pulmonary vasculature. Immune cells interact with vascular cells not only through direct contact but also by releasing cytokines and chemokines, which collectively enhance vascular contraction, endothelial dysfunction, and smooth‐muscle proliferation [211].

Among the chemokine–GPCR axes implicated in these processes, the CXCL12–CXCR4 chemokine axis mediates leukocyte recruitment, activates β‐arrestin–dependent signaling, and promotes PASMC proliferation [161]. CXCR4 inhibition mitigates vascular remodeling in preclinical models of PH [160]. This immune‐vascular crosstalk is significantly amplified under high‐altitude conditions, intensifying pulmonary vascular remodeling and contributing to the progression of right ventricular dysfunction. Another ligand–receptor pair is CCL2‐CCR2. In monocrotaline (MCT)‐induced PH, CCR2 gene deletion markedly attenuates disease severity, reducing vascular remodeling and inflammation. However, this protective effect is not observed in chronic hypoxia or Sugen5416/hypoxia (SuHx) models, suggesting that CCR2 is essential for the MCT‐driven inflammatory response but not required for other PH subtypes [212]. Notably, CCR2‐deficient mice can spontaneously develop pulmonary vascular abnormalities under normoxic conditions and display more severe PH after exposure to chronic hypoxia, likely through aberrant activation of the Notch signaling pathway [213]. To date, most evidence derives from genetic KO models, and studies using selective CCR2 inhibitors remain scarce. These findings suggest a dual role of CCR2: it promotes inflammatory remodeling under pathological stress but contributes to vascular stability under physiological conditions. Therefore, future studies should employ selective CCR2 inhibitors rather than global KO to clarify its functional contribution, and should consider model selection carefully to distinguish inflammation‐driven from hypoxia‐driven remodeling, particularly in the context of HAPH.

In addition, CXCR7 inhibitors can reduce the proliferation and remodeling of pulmonary vascular cells in neonatal mice exposed to normobaric hypoxia [214]. Other chemokine receptors such as CX3CR1 and CCR2/CCR5 have also been implicated in hypoxia‐induced vascular inflammation [215], further highlighting the contribution of immune–GPCR signaling to pulmonary vascular remodeling at high altitude.

Taken together, these findings indicate that immune‐inflammatory GPCRs, while less pronounced in HAPH than in severe or genetic PAH, likely participate in the reversible vascular remodeling characteristic of high‐altitude exposure. Future studies are needed to delineate how hypoxia and erythrocytosis modulate chemokine receptor activity, and how immune‐GPCR pathways intersect with metabolic and endothelial GPCR networks such as A_2_BR, GPER1, and S1PRs in shaping pulmonary vascular adaptation to hypoxia.

While these chemokines and inflammatory GPCRs represent well‐characterized components of vascular immune signaling, recent evidence highlights that orphan and atypical GPCRs may also contribute to pulmonary vascular remodeling under hypoxic stress. These receptors often lack clearly defined endogenous ligands but can modulate intracellular Ca^2^^+^ dynamics, cAMP signaling, and endothelial integrity—adding another regulatory layer to GPCR‐mediated pulmonary vascular adaptation.

#### 3.2.5 Orphan GPCRs in Pulmonary Vascular Remodeling

Beyond the well‐characterized vasoconstrictive, vasodilatory, and mechanosensitive GPCR pathways, emerging evidence highlights the contribution of orphan GPCRs such as GPR75 and GPR146 to hypoxia‐induced pulmonary vascular remodeling. Using complementary genetic approaches, including global KO and endothelial cell–specific conditional KO, studies demonstrate that GPR75 deficiency protects against hypoxia‐induced PH by blocking cAMP inhibition and limiting Ca^2+^‐driven contraction. Loss of GPR146 in vascular smooth‐muscle cells similarly attenuates hypoxia/SuHx‐induced PH and right ventricular (RV) hypertrophy [183, 184, 185]. Mechanistically, GPR146 drives PASMC proliferation via the 5‐LOX pathway [184] and induces endothelial pyroptosis [185], exacerbating barrier dysfunction and inflammation.

### 3.3 GPCR‐Mediated Cardiac Remodeling and Right‐Heart Dysfunction

Chronic hypoxia imposes sustained RV pressure overload, leading to maladaptive structural and functional adaptations, and ultimately HAHD. Clinically, HAHD is characterized by right ventricular enlargement and dysfunction driven by prolonged hypoxic exposure, typically in the absence of polycythemia. The pathophysiological basis lies in the interplay of PH, RV hypertrophy, and progressive cardiac dysfunction under chronic hypoxia. GPCR signaling–spanning endothelin (ET‐1/ETAR), Ang II/AT_1_R, S1PR_1_/S1PR_2_, adrenergic receptors, and chemokine receptors—integrates vasoconstriction, inflammation, and cardiac remodeling across the lung–heart axis, thereby linking chronic hypoxia to RV failure.

Chronic hypoxia leads to sustained activation of the sympathetic nervous system, which aggravates pathological cardiac remodeling. Under hypoxic stress, sustained stimulation of β‐ARs initially augments cardiac output as a compensatory mechanism but eventually drives maladaptive hypertrophy and arrhythmias [216]. Similarly, AT_1_R, as part of the renin–angiotensin system, drives myocardial fibrosis and left ventricular dysfunction via Gq–PKC and β‐arrestin pathways. These GPCR‐driven maladaptive responses contribute to right ventricular hypertrophy and progressive right‐heart failure, which constitute hallmark pathological features of HAHD.

APLNR is increasingly recognized as a cardioprotective GPCR in chronic hypoxia. Apelin‐APLNR signaling activates Gi‐dependent pathways that enhance right ventricular contractility, angiogenesis, and mitochondrial efficiency [186]. Importantly, biased agonists such as MM07 and WN561 preferentially activate G protein–mediated signaling while avoiding β‐arrestin–dependent maladaptive remodeling, thereby reducing RV hypertrophy and dysfunction [175, 177]. These agents hold translational promise for mitigating HAHD progression.

Sex hormone signaling, particularly estrogenic pathways, exerts a major influence on cardiac adaptation to chronic hypoxic exposure. Estrogen mediates cardioprotective effects through GPER1, suppresses HIF‐1α and macrophage migration inhibitory factor expression in hypobaric hypoxia models, thereby attenuating myocardial inflammatory responses and reduces susceptibility to arrhythmogenesis [187]. These pathways may contribute to the reduced susceptibility to HAHD in premenopausal women compared to men and postmenopausal women. Consequently, pharmacological targeting of the estrogen–GPER1 axis represents a potential sex‐specific therapeutic strategy for HAHD.

## 4 Preclinical In Vivo Models of Acute and Chronic High‐Altitude Diseases

Animal models serve an indispensable role in investigating both acute and chronic HADs. Through the use of standardized animal models, researchers have achieved significant advances in understanding the underlying mechanisms of altitude‐related disorders and in developing effective prevention and treatment approaches. Currently, two primary methodologies are employed for constructing animal models of HADs. The first approach utilizes high‐altitude environmental simulation chambers that precisely replicate the low‐pressure, oxygen‐depleted conditions found at specific elevations by carefully controlling the atmospheric pressure within the chamber [217, 218, 219]. The second method involves directly transporting experimental animals to actual high‐altitude locations ranging from 3000 to 5000 m above sea level [220]. Each modeling approach offers distinct advantages and limitations. Environmental simulation chambers provide precise control over atmospheric pressure and oxygen levels, while allowing researchers to incorporate additional environmental factors such as ultraviolet radiation exposure, low temperatures, and high wind velocities. This method offers operational convenience and excellent reproducibility, making it the preferred approach in contemporary high‐altitude medical research. Nevertheless, simulation chambers cannot fully replicate the complete spectrum of conditions encountered in genuine high‐altitude environments. Conversely, on‐site high‐altitude modeling provides the most comprehensive representation of real‐world high‐altitude conditions, offering superior environmental authenticity. However, this approach faces practical challenges including geographical limitations, elevated costs, and increased difficulty in maintaining controlled experimental conditions.

### 4.1 In Vivo Models of Acute Hypoxia Exposure

Animal models of AHAD are primarily used to simulate key pathological features associated with AMS, HAPE, and HACE, although they cannot fully recapitulate the complete clinical syndromes observed in humans [221, 222]. In practice, these models are usually established by rapid exposure to severe hypobaric hypoxia over hours to several days [221, 223]. Among them, HAPE models are most commonly produced in rats by exposure to simulated altitudes around 5000–6000 m, sometimes combined with exercise to aggravate pulmonary vascular stress and promote edema formation [224]. Outcome measures typically include lung wet‐to‐dry ratio, bronchoalveolar protein concentration, pulmonary histopathology, and blood‐gas abnormalities [224]. HACE models are more difficult to reproduce with hypoxia alone, and some protocols therefore combine hypobaric hypoxia with additional insults, such as temperature fluctuation or inflammatory stimulation, to enhance BBB disruption and cerebral edema [225]. In contrast, a fully satisfactory animal model of AMS remains difficult to define because hallmark human symptoms such as headache and dizziness cannot be directly assessed in animals; therefore, current AMS‐related models usually focus on acute hypoxic injury and associated neurobehavioral or organ‐level responses rather than on a strict reproduction of the clinical syndrome itself [222].

### 4.2 In Vivo Models of Chronic Hypoxia Exposure

CHAD models are primarily used to mimic CMS or HAPC resulting from prolonged hypobaric hypoxia [226, 227]. These models generally require exposure for weeks to months rather than days [226]. Rodents, especially rats, are commonly subjected to intermittent or continuous hypobaric hypoxia at simulated altitudes of approximately 4500–5500 m, leading to progressive increases in hemoglobin concentration, hematocrit, erythrocyte count, blood viscosity, PH, and right‐heart strain [226, 228]. Some rat strains are particularly susceptible and develop a phenotype resembling human CMS, including severe polycythemia, hypoxemia, and cardiopulmonary maladaptation [227]. Compared with acute models, chronic models are better suited for studying long‐term maladaptation to hypoxia, but they are more time‐consuming and are influenced more strongly by strain, exposure schedule, and chamber protocol [226].

Importantly, these in vivo systems provide the experimental basis for validating GPCR‐dependent mechanisms and for testing whether pathway‐directed interventions can modify disease susceptibility, progression, or reversibility. They also provide a useful platform for evaluating disease‐stage‐specific GPCR targets and for linking molecular mechanisms to physiological outcomes relevant to HADs.

## 5 GPCR‐Targeted Therapeutic Strategies for High‐Altitude Diseases

HADs comprise a spectrum of acute and chronic disorders that, despite heterogeneous clinical manifestations, converge on several shared pathogenic processes, including dysregulated vascular tone, endothelial and epithelial barrier disruption, inflammation, erythropoietic imbalance, and cardiopulmonary remodeling. Across these processes, GPCRs provide a druggable signaling framework that links hypoxic stress to organ‐specific maladaptation. Therefore, therapeutic strategies for HADs can be viewed not only as supportive interventions aimed at correcting hypoxemia, but also as opportunities to modulate GPCR‐mediated hypoxic responses in a disease‐stage‐specific manner.

### 5.1 Current Supportive and Standard Therapeutic Approaches

Current management of HADs remains primarily centered on supportive and symptomatic strategies. The most direct and effective approaches for treating both acute and chronic altitude sickness involve descending to lower elevations and implementing standardized oxygen therapy protocols. Diffuse oxygen supply systems in high‐altitude living environments have emerged as a crucial strategy for managing these conditions [229]. In pharmaceutical treatment, Tibetan medicinal extracts, particularly *Rhodiola rosea* and corticosteroid compounds, have demonstrated promising efficacy in reducing acute high‐altitude sickness, with related formulations now clinically validated and widely implemented [230, 231, 232].

For chronic or recurrent hypoxia‐related conditions, treatment is often directed toward improving oxygenation, reducing cardiopulmonary burden, and managing disease‐specific complications. For example, in obstructive sleep apnea syndrome among high‐altitude residents, randomized double‐blind placebo‐controlled evidence indicates that acetazolamide can reduce 24‐h mean arterial blood pressure, decrease nocturnal respiratory events, and improve nighttime oxygen saturation [233]. Nevertheless, most currently available interventions remain supportive, preventive, or symptomatic rather than mechanism‐directed. This limitation has prompted increasing interest in GPCR‐targeting strategies, because GPCRs regulate many of the core pathophysiological processes that underlie HADs, including ventilatory adaptation, cerebral and pulmonary vascular tone, alveolar fluid clearance, vascular remodeling, inflammation, and erythropoietic responses.

### 5.2 Repurposed GPCR‐Targeting Agents for Acute High‐Altitude Diseases

Several clinically available GPCR‐targeting drugs have been explored as repurposed agents for AHADs, especially AMS, HACE, and HAPE. These agents do not replace first‐line interventions such as descent and oxygen supplementation, but they may provide adjunctive benefits by modulating disease‐relevant GPCR pathways involved in nausea, headache, cerebral vascular tone, intracranial pressure, and alveolar fluid clearance.

For AMS and high‐altitude headache symptom management, dopamine D_2_ receptor antagonists and serotonin 5‐HT_1_B/_1_D receptor agonists are of particular interest. Dopamine D_2_ receptor antagonists, such as metoclopramide, have been used to alleviate nausea and headache associated with AMS and HACE, in part through effects on dopaminergic regulation of cerebral vascular tone and intracranial pressure. A prospective double‐blind randomized field trial in the Everest region demonstrated that a single 10 mg oral dose of metoclopramide provided symptom relief comparable to ibuprofen, with a more pronounced reduction in nausea [72]. However, its use remains off‐label and is not incorporated into formal clinical guidelines due to limited sample size and marginal improvement in overall AMS scores.

Serotonergic modulation represents another repurposed strategy for acute high‐altitude headache and AMS prevention. In a randomized controlled field trial (ISRCTN87201238), 5‐HT_1_B/_1_D receptor agonist sumatriptan was evaluated for AMS prophylaxis and acute high‐altitude headache treatment. This prophylactic trial demonstrated a significantly lower incidence of AMS in the sumatriptan group compared with placebo [79]. Nevertheless, the clinical application of triptans in high‐altitude medicine remains limited by the small number of trials, potential cardiovascular safety concerns, and the lack of broad guideline endorsement.

For HAPE, β‐AR agonist salmeterol has been investigated because of their ability to enhance alveolar fluid clearance and has been associated with a reduced risk of HAPE. A case report documented successful secondary prevention of HAPE using a combination of sildenafil, salmeterol, and acetazolamide in a Mount Everest summiteer with prior HAPE [234], and a Phase 1/2a trial (NCT06040268) is currently evaluating the salmeterol/fluticasone combination under simulated hypoxia conditions in both healthy individuals and HAPE‐susceptible subjects. Nevertheless, the evidence supporting salmeterol for HAPE prevention remains limited and has not been validated in large‐scale randomized controlled trials [235]. Therefore, salmeterol should currently be regarded as an adjunctive or investigational preventive option rather than a standard replacement for established HAPE management.

### 5.3 GPCR‐Targeted Strategies for Chronic High‐Altitude Cardiopulmonary Disease

In CHADs, particularly HAPH and HAHD, GPCR‐targeting strategies mainly focus on pulmonary vascular tone, vascular remodeling, and right ventricular stress. Unlike AHADs, in which GPCR‐targeting agents are mainly used for symptom relief or short‐term prevention, chronic hypoxia‐associated cardiopulmonary disorders require interventions that modulate sustained vasoconstriction, vascular remodeling, and pressure overload.

In chronic hypoxia‐associated disorders, including HAPH and HAHD, AT_1_R antagonists and ETR antagonists (e.g., bosentan and macitentan) have demonstrated potential improvements in PAP and indices of vascular remodeling. Among these agents, ETR antagonists represent one of the most clinically relevant GPCR‐targeting strategies, because endothelin signaling directly contributes to hypoxia‐induced pulmonary vasoconstriction and vascular remodeling. Bosentan was evaluated in a Phase 2 trial (NCT00260819) and shown in a preliminary crossover study to reduce hypoxia‐induced pulmonary artery systolic pressure in both healthy individuals and HAPE‐susceptible subjects [88]. However, prophylactic administration over 5 days prior to ascent failed to improve exercise capacity or lower pulmonary pressure in healthy subjects and was associated with worsened oxygen saturation during exercise [236]. These inconsistent findings suggest that ETR blockade may be beneficial only in selected physiological or disease contexts, rather than as a broadly applicable prophylactic strategy for all individuals exposed to high altitude.

Additionally, a Phase 1 randomized, double‐blind, four‐sequence crossover study (NCT01794078) evaluated the safety of combined aminophylline (an AR antagonist) and ambrisentan (a selective ETAR antagonist) during exercise at simulated high altitude in healthy volunteers. This combination strategy is mechanistically attractive because it targets distinct GPCR pathways involved in hypoxic vascular tone and exercise responses. However, its clinical relevance for established HAPH or HAHD remains uncertain, as the available evidence is still limited to simulated high‐altitude conditions and selected study populations.

These agents remain investigational or adjunctive in high‐altitude medicine, and their integration into standard care is constrained by the lack of robust trial data and guideline endorsement [1, 237]. Therefore, future clinical studies should distinguish between healthy high‐altitude exposure, HAPE susceptibility, established HAPH, and HAHD, because these conditions may differ substantially in disease stage, vascular remodeling burden, right ventricular involvement, and responsiveness to GPCR‐targeted interventions. To integrate these disease‐context‐specific considerations, Figure 5 summarizes the major GPCR‐targeted therapeutic strategies across acute and CHADs, highlighting their associated pathological modules, representative receptor targets, translational maturity, and genetic susceptibility factors.

**FIGURE 5:**
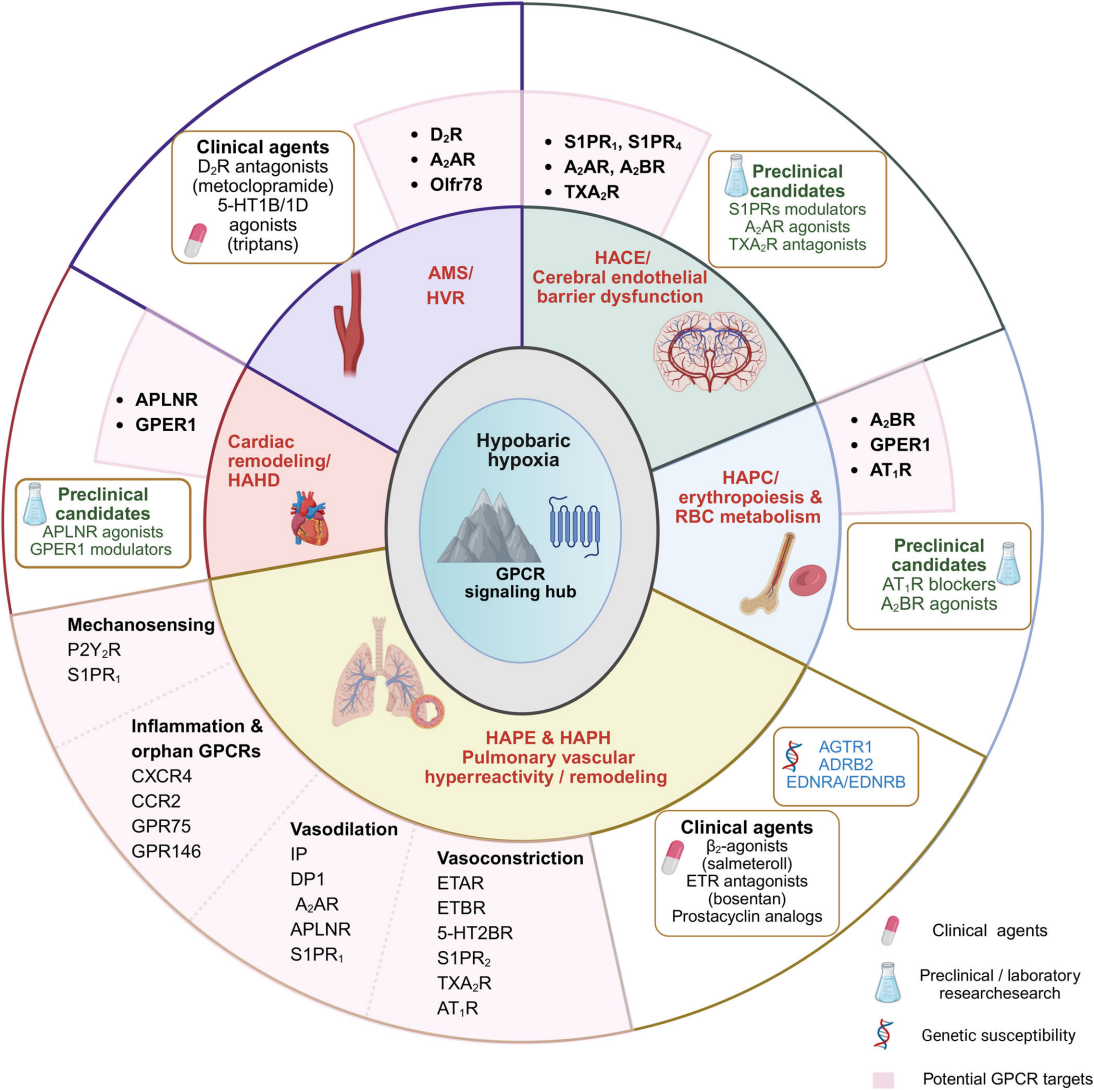
GPCR‐targeted therapeutic landscape and genetic susceptibility across high‐altitude diseases. Hypobaric hypoxia acts as a central stimulus that activates multiorgan GPCR signaling networks across acute and chronic high‐altitude diseases. The inner ring summarizes major disease‐related pathological modules, including HVR in AMS, cerebral endothelial barrier dysfunction in HACE, erythropoiesis and RBC metabolism in HAPC, pulmonary vascular hyper‐reactivity and remodeling in HAPE and HAPH, and cardiac remodeling in HAHD. Representative GPCR targets are shown within each module. Clinically available or repurposed agents include dopamine D_2_ receptor (D_2_R) antagonists such as metoclopramide, 5‐hydroxytryptamine 1B/1D (5‐HT_1_B/_1_D) receptor agonists such as triptans, β_2_‐adrenergic receptor agonists, endothelin receptor antagonists, and prostacyclin analogs. Preclinical candidates include sphingosine‐1‐phosphate receptor (S1PR) modulators, adenosine A2A receptor (A_2_AR) agonists, thromboxane A2 receptor (TXA_2_R) antagonists, apelin receptor (APLNR) agonists, GPER1 modulators, AT1R blockers, and A2BR agonists. In the pulmonary vascular module, GPCR pathways are further grouped into mechanosensing, inflammatory and orphan GPCR signaling, vasodilation, and vasoconstriction. Genetic susceptibility factors, including angiotensin II Type 1 receptor gene (AGTR1), ADRB2, and endothelin receptor type A/B gene (*EDNRA*/*EDNRB*), may contribute to interindividual differences in high‐altitude disease risk and therapeutic responsiveness. Overall, this framework highlights the receptor subtype‐, organ‐, and disease‐stage‐specific nature of GPCR signaling and supports precision GPCR‐targeted strategies for high‐altitude diseases.

### 5.4 Emerging and Preclinical GPCR Targets

Beyond established clinical use, accumulating experimental and translational studies indicate that GPCR pathways function as upstream regulators of hypoxia‐induced vascular, inflammatory, and metabolic responses. These emerging targets are not yet part of routine HAD management, but they provide a mechanistic basis for developing receptor‐selective, disease‐stage‐specific, and organ‐targeted interventions.

Adenosine A_2_AR/A_2_BR regulate vascular permeability and inflammatory signaling, while sphingosine‐1‐phosphate receptors exert divergent effects, with S1PR1 and S1PR4 supporting BBB integrity and S1PR_2_ promoting pulmonary vascular remodeling. This receptor subtype divergence suggests that therapeutic strategies should aim to enhance protective GPCR signaling while suppressing maladaptive receptor pathways, rather than broadly activating or inhibiting an entire ligand system. APLNR signaling contributes to right ventricular adaptation under hypoxic stress, and G protein–biased APLNR agonists (e.g., MM07 and WN561) have shown cardioprotective effects in preclinical models. These findings also highlight biased agonism as a promising pharmacological approach for preserving beneficial GPCR signaling while minimizing adverse downstream effects in chronic hypoxia.

Additional GPCRs, including GPER1, CXCR4, GPR75, and GPR146, have been implicated in sex‐specific susceptibility, immune–metabolic regulation, and inflammatory remodeling, although their roles in HADs remain under active investigation. Although these targets are biologically plausible, most remain at the mechanistic or preclinical stage. Their therapeutic relevance in HADs will require validation in disease‐specific animal models, human tissue studies, and carefully designed clinical cohorts.

### 5.5 Precision Medicine and Future Challenges

Importantly, accumulating candidate gene studies suggest that genetic polymorphisms in several GPCRs may contribute to interindividual susceptibility to HADs. This genetic dimension links emerging GPCR pharmacology with precision medicine, as receptor variants can influence both disease risk and therapeutic responsiveness. Among these, ADRB2 is one of the most consistently implicated genes. Functional variants such as rs1042713 (Arg16Gly) and rs1042714 (Gln27Glu) have been associated with susceptibility to HAPE [238, 239, 240]. Given the established role of β_2_AR signaling in alveolar fluid clearance, as discussed above, ADRB2 genotype may partly explain interindividual differences in HAPE susceptibility and may help identify individuals who are more likely to benefit from β_2_AR‐targeted strategies. However, this pharmacogenomic hypothesis still requires prospective validation.

In addition, genetic variation in other GPCR pathways involved in vascular regulation has also been reported. Studies on polymorphisms in AGTR1 have reported mixed associations with HAPE susceptibility, highlighting the need for further validation in larger cohorts [90, 241].

Sex differences represent another important dimension of precision GPCR pharmacology in HADs. As discussed above, estrogen‐related signaling through membrane‐associated GPER1 may intersect with HIF signaling, erythropoietic regulation, endothelial function, vascular inflammation, and cardiac adaptation under hypoxia. These mechanisms may contribute to sex‐related differences in susceptibility to CMS, pulmonary vascular remodeling, and right ventricular adaptation. Therefore, future studies should incorporate sex‐stratified analyses of GPCR expression, genetic variants, circulating biomarkers, and treatment responses. In this context, the estrogen–GPER1 axis may serve as a potential framework for developing sex‐informed GPCR‐targeted strategies, although its disease‐specific relevance in HADs requires further validation.

Biomarker‐guided stratification may further strengthen precision GPCR targeting in HADs. Because different GPCR pathways dominate distinct disease phenotypes and stages, circulating and physiological biomarkers could help identify the most relevant receptor axes for intervention. For example, elevated ET‐1 levels, increased PAP, or exaggerated HPV may indicate activation of the ETR pathway; impaired alveolar fluid clearance, reduced β_2_AR responsiveness, or risk‐associated ADRB2 genotypes may support investigation of β_2_AR‐targeted strategies in selected HAPE‐prone individuals; and increased adenosine, S1P, CXCL12, or inflammatory chemokines may reflect activation of adenosine, S1P, or chemokine receptor pathways. In chronic high‐altitude exposure, hemoglobin concentration, hematocrit, EPO levels, oxygen saturation, and erythrocyte metabolic markers such as 2,3‐BPG or S1P may help distinguish adaptive from maladaptive hematologic responses. Although these biomarkers are not yet established as clinical decision tools, they may provide a framework for patient stratification, pathway selection, and treatment‐response monitoring in future GPCR‐targeted trials.

## 6 Conclusions and Perspectives

HADs arise from insufficient adaptation to hypobaric hypoxia and encompass a continuum from acute hypoxic stress to chronic maladaptive remodeling. Across this disease spectrum, GPCRs act as key molecular integrators that translate hypoxic, metabolic, inflammatory, neurohumoral, and mechanical cues into organ‐specific responses. By regulating ventilatory control, pulmonary and cerebral vascular tone, endothelial and epithelial barrier integrity, erythropoiesis, immune activation, pulmonary vascular remodeling, and right‐heart adaptation, GPCR networks provide a unifying mechanistic framework for understanding the progression of HADs.

A major concept emerging from current evidence is that GPCR signaling in HADs is highly context dependent. The same ligand system may exert protective or pathogenic effects depending on receptor subtype, cell type, disease stage, and duration of hypoxic exposure. For example, adenosine, sphingosine‐1‐phosphate, adrenergic, endothelin, prostaglandin, chemokine, apelin, and estrogen‐related GPCR pathways may contribute to either adaptive compensation or maladaptive injury under different conditions. This receptor‐ and stage‐specific divergence highlights the need to move beyond broad pathway modulation toward precise targeting of disease‐relevant GPCR subtypes.

Despite substantial progress, several important challenges remain. Many GPCR‐related mechanisms have been identified primarily in cellular systems or animal models, whereas direct validation in human high‐altitude populations remains limited. In addition, AHADs and CHADs differ markedly in onset, dominant pathological processes, reversibility, and therapeutic windows. Genetic background, ethnicity, sex, altitude of residence, ascent profile, cold exposure, and comorbidities may further influence GPCR signaling and treatment responsiveness. Future studies should therefore integrate disease‐stage‐specific models, multiethnic human cohorts, standardized phenotyping, and clinically relevant outcome measures.

Looking forward, GPCR‐targeted strategies may offer new opportunities for precision intervention in HADs. Priority areas include receptor subtype‐specific modulation, biased agonism, tissue‐selective drug delivery, pharmacokinetic and pharmacodynamic assessment under hypobaric hypoxia, and biomarker‐guided patient stratification. Integrating GPCR pharmacology with genomics, metabolomics, and physiological monitoring may help distinguish adaptive from maladaptive hypoxic responses and identify individuals most likely to benefit from targeted therapy. Ultimately, defining how GPCR networks govern high‐altitude adaptation and maladaptation may support the development of individualized preventive and therapeutic strategies for high‐altitude populations.

## Author Contributions

*Conceptualization*: Qing Zhao, Kun Liang, Huaping Zheng, Cheng Deng, and Fengming Luo. *Literature search and analysis*: Qing Zhao, Lu Dai, Kun Zheng, and Kun Liang. *Methodology*: Qing Zhao, Lu Dai, Kun Zheng, Ling Chen, Xuefei He, Hui Yan, and Lei Chen. *Investigation and clinical interpretation*: Changliang Liu, Lianhua Liu, Chuanling Pan, Ling Chen, Xuefei He, Hui Yan, and Lei Chen. *Writing – original draft*: Qing Zhao, Lianhua Liu, Lu Dai, and Kun Zheng. *Writing – review and editing*: Qing Zhao, Changliang Liu, Lu Dai, Kun Zheng, Kun Liang, Chuanling Pan, Lianhua Liu, Ling Chen, Xuefei He, Hui Yan, Lei Chen, Huaping Zheng, Cheng Deng, and Fengming Luo. *Visualization*: Qing Zhao and Lu Dai. *Supervision*: Huaping Zheng, Cheng Deng, and Fengming Luo. *Funding acquisition*: Huaping Zheng, Cheng Deng, and Fengming Luo. *Project administration*: Huaping Zheng. All the authors critically reviewed the manuscript, contributed to scientific discussions, and approved the final version of the manuscript and the response to reviewers.

## Ethics Statement

The authors have nothing to report.

## Conflicts of Interest

The authors declare no conflicts of interest.

## Data Availability

The authors have nothing to report.
